# The contribution of extra‐pair paternity to the variation in lifetime and age‐specific male reproductive success in a socially monogamous species

**DOI:** 10.1111/evo.14473

**Published:** 2022-04-09

**Authors:** Sara Raj Pant, Maaike A. Versteegh, Martijn Hammers, Terry Burke, Hannah L. Dugdale, David S. Richardson, Jan Komdeur

**Affiliations:** ^1^ Groningen Institute for Evolutionary Life Sciences, Faculty of Science and Engineering University of Groningen Groningen The Netherlands; ^2^ Centre for Ecology, Evolution and Conservation, School of Biological Sciences University of East Anglia, Norwich Research Park Norwich UK; ^3^ Department of Biology Lund University Lund Sweden; ^4^ Aeres University of Applied Sciences Almere The Netherlands; ^5^ Department of Animal and Plant Sciences University of Sheffield Sheffield UK; ^6^ Nature Seychelles Roche Caiman Mahe Republic of Seychelles

**Keywords:** Age‐specific reproduction, extra‐pair paternity, lifetime reproductive success, opportunity for selection, sexual selection, Seychelles warbler

## Abstract

In socially monogamous species, extra‐pair paternity (EPP) is predicted to increase variance in male reproductive success (RS) beyond that resulting from genetic monogamy, thus, increasing the “opportunity for selection” (maximum strength of selection that can act on traits). This prediction is challenging to investigate in wild populations because lifetime reproduction data are often incomplete. Moreover, age‐specific variances in reproduction have been rarely quantified. We analyzed 21 years of near‐complete social and genetic reproduction data from an insular population of Seychelles warblers (*Acrocephalus sechellensis*). We quantified EPP's contribution to lifetime and age‐specific opportunities for selection in males. We compared the variance in male genetic RS *vs* social (“apparent”) RS (RS_ap_) to assess if EPP increased the opportunity for selection over that resulting from genetic monogamy. Despite not causing a statistically significant excess (19%) of the former over the latter, EPP contributed substantially (27%) to the variance in lifetime RS, similarly to within‐pair paternity (WPP, 39%) and to the positive WPP‐EPP covariance (34%). Partitioning the opportunity for selection into age‐specific (co)variance components, showed that EPP also provided a substantial contribution at most ages, varying with age. Therefore, despite possibly not playing the main role in shaping sexual selection in Seychelles warblers, EPP provided a substantial contribution to the lifetime and age‐specific opportunity for selection, which can influence evolutionary processes in age‐structured populations.

Extra‐pair paternity (EPP), obtained by mating outside the pair bond, is common across socially monogamous species (Uller and Olsson 2008; Leclaire et al. 2013; Lee‐Jenkins et al. 2015; Dillard 2017; Brouwer and Griffith 2019). It has been widely hypothesized that higher‐quality males are more successful at gaining both EPP and within‐pair paternity (WPP; Jennions and Petrie 2000; Hsu et al. 2015). Consequently, EPP is predicted to covary positively with WPP and increase the total reproductive output of males with an already high WPP (Jennions and Petrie 2000; Ackay and Roughgarden 2007). Therefore, EPP should increase the variance in male total reproductive success (RS) beyond that arising from a genetically monogamous mating system (Webster et al. 1995). This increase in the variance of RS translates into an EPP‐mediated rise in the “opportunity for selection”, i.e., the maximum strength of selection that can act on a trait (Arnold and Wade 1984). The opportunity for selection is estimated as the mean‐standardised variance in RS, i.e., the ratio of the variance in RS to the squared mean RS (Arnold and Wade 1984).

Many studies have investigated whether EPP increases the opportunity for selection in socially monogamous systems. Such studies, which have been mainly conducted in avian species, compared the standardized variance in genetic RS (which we refer to simply as “RS”), i.e., the total number of offspring sired by a male (WPP + EPP), to the standardized variance in social (“apparent”) reproduction (RS_ap_), i.e., the number of offspring produced by a male's social female and not necessarily sired by the male (reviewed in: Lebigre et al. 2012). Several of these studies also assessed the contributions of EPP, WPP, and their covariance to the standardized variance in male RS. The covariance between EPP and WPP is often considered because, depending on its sign, EPP may increase (positive covariance) or decrease (negative covariance) the opportunity for selection (Webster et al. 1995, 2007; Lebigre et al. 2012). However, the evidence that EPP increases the opportunity for selection is mixed. While several studies have shown a large increase (e.g., Yezerinac et al. 1995; Kleven et al. 2006; Albrecht et al. 2007; Dolan et al. 2007), others have found only a limited or no change in the opportunity for selection via EPP (e.g., Webster et al. 2001, 2007; While et al. 2011; Lebigre et al. 2012; Grunst et al. 2019). Studies assessing whether genetic promiscuity increases the opportunity for selection among males in different social mating systems (including monogamy, polygyny and multimale groups with one or more females) also provided variable results (see Weatherhead and Boag 1997 [birds]; Jones et al. 2001 [fish]; Collet et al. 2012 [birds]; Isvaran and Sankaran 2017 [mammals]). Interestingly, the effect of promiscuity on the opportunity for selection can be modulated by the type of social mating system, with extra‐pair or extra‐group paternity (EGP) increasing the opportunity for selection under no or low social polygyny and decreasing it in highly polygynous mating systems (Isvaran and Sankaran 2017). More research, across different taxonomic groups, based on complete social and genetic data is needed to avoid common pitfalls (see following paragraphs) and better understand how EPP may impact the opportunity for selection in natural populations.

Investigating whether and how EPP increases the variance in RS is challenging in wild populations. First, studies are often unable to assign paternity to a large fraction of extra‐pair offspring, often because they work on open study systems, where sampling all males and their offspring is not possible. This is problematic because incomplete sampling of extra‐pair sires and of the young produced by individual males causes a systematic underestimation of the mean RS. Given that the opportunity for selection is the mean‐standardized variance in RS (estimated as $\frac{\mathrm{variance}\;\mathrm{RS}}{{\left(\mathrm{mean}\;\mathrm{RS}\right)}^{2}}$), underestimating the mean RS leads to an overestimation of the opportunity for selection and, consequently, of the effect of EPP on it (Freeman‐Gallant et al. 2005; Lebigre et al. 2012). Second, it is often not possible to perform random sampling of males in a population with respect to genetic and/or phenotypic quality, as it is often only possible to sample successful reproducers (i.e., high‐quality males). This can be due to the relative ease in detecting successful males that defend their territories/social females and rear dependent offspring, compared to males that do not obtain a territory/social mate or compared to males whose breeding attempt(s) fail. This bias may cause a significant underestimation of males with zero RS and a consequent bias in the mean and variance of RS (Webster et al. 2001, 1995; Sheldon and Ellegren 1999; Lebigre et al. 2012; Schlicht and Kempenaers 2013). Third, the standardized variance in RS is often measured within a single year, or at best, over a few years. This is problematic, because it is lifetime, rather than annual, RS that constitutes the genetic contribution of an individual to the next generation (Brommer et al. 2002). Therefore, the standardized variance in lifetime RS better captures the total opportunity for selection that ultimately shapes evolutionary processes (Brommer et al. 2002; Lebigre et al. 2012).

To our knowledge, the standardized variance in lifetime genetic RS (LRS) and lifetime social (“apparent”) RS (LRS_ap_) has been estimated only in three socially monogamous species (avoiding the other mentioned pitfalls), i.e., splendid fairy‐wren, *Malurus splendens* (study period: 7 years, *n*: 204 males) (Webster et al. 2007), song sparrow, *Melospiza melodia* (study period: 16 years, *n*: 183 males) (Lebigre et al. 2013, 2012) and white‐throated sparrow, *Zonotrichia albicollis* (study period: 17 years, *n*: 277 males) (Grunst et al. 2019). These studies have not detected a substantial difference between the standardized variance in LRS and LRS_ap_. Only one of these studies (Lebigre et al. 2013) also quantified the age‐specific standardized variances in RS and RS_ap_ (and found considerable variation across ages), despite this being important in age‐structured populations (Coulson and Tuljapurkar 2008).

In iteroparous species, there is ample evidence for changes across different ages in mean RS, which often increases in early life and declines in late life (see reviews of Nussey et al. 2013; Lemaître and Gaillard 2017).  A large body of work also provides evidence that both EPP and WPP, which together form RS, vary with age, as older males (up to the point of senescence) are generally found to gain more EPP and lose less WPP (e.g., Cleasby and Nakagawa 2012; Hsu et al. 2015, 2017; Raj Pant et al. 2020). If, in addition to the mean, the variance in age‐specific EPP were to differ across male age groups, this may cause the variance in RS, and thus, the opportunity for selection, to vary with age. These changes may, in turn, affect demographic variance (i.e., variance in survival and reproduction of individuals within a population in a year; Engen et al. 2005b), genetic drift, and inbreeding (Arnold and Wade 1984; Engen et al. 2005a; Vindenes et al. 2008; Lebigre et al. 2013). Studies solely addressing the overall standardized variance in LRS overlook the effect that EPP may have at different ages on both the age‐specific and total (lifetime) opportunity for selection. Comprehensive analyses that quantify both the lifetime and age‐specific standardized variances in RS, and compare such variances with the standardized variances in lifetime and age‐specific RS_ap_, are required if we want to better understand the effects of EPP on evolutionary processes in age‐structured populations (Lebigre et al. 2013).

Here, we analyses 21 years (1997–2018) of genetic pedigree and life‐history data for 237 males from a natural population of Seychelles warblers (*Acrocephalus sechellensis*) on Cousin Island, Seychelles. This species is a facultative cooperative‐breeder and is socially monogamous but genetically promiscuous (Komdeur 1992; Richardson et al. 2001). Circa 44% of young are sired by males other than a female's social male, and this sire is almost always an extra‐group male, i.e., a (dominant) male from another territory (Richardson et al. 2001; Hadfield et al. 2006). Seychelles warblers are long‐lived (mean life expectancy: 5.5 years after fledging, maximum observed lifespan: 19 years; Komdeur 1991; Hammers and Brouwer 2017) and undergo a senescent decline in survival after 6 years of age (Hammers et al. 2013). Reproduction is also age‐dependent: in particular, in males, annual within‐group paternity (WGP) and EGP acquisition display an early‐life increase and a late‐life decline (Raj Pant et al. 2020). Moreover, between‐individual differences in annual WGP and EGP are unrelated to selective appearance and disappearance (Raj Pant et al. 2020). Accurate data on EPP are available because inter‐island migration is virtually absent (Komdeur et al. 2004) and, since 1997, over 96% of birds have been individually color‐ringed and blood sampled (for molecular sexing and parentage assignment), with their annual RS monitored from birth till death (Brouwer et al. 2010; Hammers et al. 2015). Using these data, which allows us to avoid the pitfalls described earlier, we: (I) quantify the total and age‐specific opportunity for selection via EGP and (II) assess whether EGP increases the amount of standardized variance in male RS beyond that arising under the apparent (social) mating system in the Seychelles warbler. For this, we estimate the contribution of EGP to the mean‐standardized variance in lifetime and age‐specific RS. We then compare the mean‐standardized lifetime and age‐specific variances in RS *vs* RS_ap_.

## Methods

### STUDY SYSTEM

The Seychelles warbler is an insectivorous passerine endemic to the Seychelles archipelago. The population on Cousin Island (29 ha, 04°20′S, 55°40′E) has been monitored as part of a long‐term study, which started in 1981 and was intensified from 1997 (Komdeur 1992; Richardson et al. 2003; Hammers et al. 2019). Virtually all (successful and unsuccessful) breeding events have been followed each year during the major breeding season (June–September) and, often, also during the minor breeding season (January–March; Hammers et al. 2019). Every year, as many individuals as possible were caught, either in the nest (nestlings) or using mist nets (recently fledged juveniles and adults). Newly caught birds were assigned a unique combination of three color rings and a British Trust for Ornithology metal ring. DNA extracted from blood samples was used for molecular sexing (following Griffiths et al. 1998) and genotyping based on 30 microsatellite loci (Richardson et al. 2001; Spurgin et al. 2014). This enabled the creation of a pedigree for the population, with parentage assigned to 2039 individuals (born 1991–2018) using MasterBayes 2.52 (methods in: Hadfield et al. 2006; Edwards et al. 2018; Sparks et al. 2021). The population is effectively a “closed system” with virtually inter‐island dispersal (<0.1%; Komdeur et al. 2016, 2004) and very high resighting probability (98%; Brouwer et al. 2010). This enables us to monitor virtually all individuals throughout their lives and to accurately calculate parentage, survival, and RS (annual and lifetime).

Seychelles warblers are facultatively cooperative breeders and territorial. They live in and defend territories that are more or less stable (spatially and temporally) as pairs or in groups of three to eight individuals (*ca* 50% of territories have >2 individuals; Hammers et al. 2019). Groups consist of a dominant breeding pair, which live in the same territory until death, sexually mature subordinates, and dependent young of either sex (Komdeur 1992; Richardson et al. 2002; Kingma et al. 2016a). Each season, group membership and individual social status were assigned to all birds. Groups and their territory boundaries were identified using observations of foraging and singing locations, nonaggressive social interactions, and aggressive territorial interactions (e.g., Bebbington et al. 2017). Within groups, dominant pairs were identified via pair and courtship behaviors. Subordinate birds, which are often, but not always, offspring that have delayed dispersal (Komdeur 1992; Kingma et al. 2016a) were assigned “helper” or “non‐helper” status based on whether they contributed to raising young in their territory (Komdeur 1994; Richardson et al. 2002).

Seychelles warblers are socially, but not genetically, monogamous and *ca* 44% of offspring are sired by males other than the dominant male in their group (Richardson et al. 2001; Hadfield et al. 2006; Raj Pant et al. 2020). Clutches typically consist of one egg, though *ca* 20% of nests contain one or two extra eggs, often laid by subordinate females (11% of offspring in the population are produced by subordinate females; Richardson et al. 2001; Hammers et al. 2019; Raj Pant et al. 2019). Over 97% of all paternity is acquired by dominant males (Richardson et al. 2001; Raj Pant et al. 2019) either in their own territory (WGP) or with females living in other territories (EGP). For simplicity, we refer to dominant females and the cobreeding subordinate females as the “social females” of the dominant male in their group, because dominant males can produce offspring with both the dominant and subordinate female(s) in their territory (Richardson et al. 2002, 2001). However, in most cases, breeding is carried out by the dominant pair alone (89% of offspring are produced by dominant females; Raj Pant et al. 2019). About 70% (1997–1999) or 50% (2003–2014) of territories are occupied only by the dominant pair, while in the remainder territories, the pair is joined by subordinates of either sex (Kingma et al. 2016a). On multifemale territories, only about 40% of helper females breed, producing ca. 40% of the offspring of these territories (Richardson et al. 2001; Hammers et al. 2019). Moreover, dominant males form a life‐long pair bond with dominant, but not subordinate, females; further, the bond between dominant individuals is tighter, with dominant pairs exhibiting clear courtship and pair behaviors and males mostly mate‐guarding dominant females even when subordinate females are present in the group (Komdeur et al. 1999). Hence, the Seychelles warbler system can be considered as a special case of social monogamy. In the Seychelles warbler, there is evidence that dominant males seek EGP via extraterritorial forays (Komdeur et al. 1999) and 59% of extra‐group offspring are sired by males from within two territories away (Richardson et al. 2001). The risk of WGP loss suffered by dominant males is known to be higher in larger groups and to be unrelated to clutch size and several other socio‐ecological factors including breeding density, breeding synchrony, and territory quality (Raj Pant et al. 2019).

### DATASET ASSEMBLY AND DESCRIPTION

We compiled a dataset (spanning 21 years: 1997–2018) of 237 reproductively mature males born on Cousin in 1997–2005, for which complete data on annual and lifetime RS (i.e., the total number of offspring produced in life) were available. This dataset does not include 33 individuals who were translocated in 2004 and 2011 to other islands for conservation reasons (Richardson et al. 2006; Wright et al. 2014). It also excludes six individuals who had not died by 2018 (i.e., the last year with pedigree data). The upper bound of 2005 for hatch year was set to avoid biasing the dataset toward short‐lived males born in later years of the field project. Individuals were assigned their age in years, rounded up to the closest integer (e.g., an individual was assigned the age of 3 when they were ≥2.5 and <3.5 years old). Since reproductive maturity occurs at around 8 months, we assigned age of 1 year to all males aged ≥8 months and <1.5 years. Parentage data for young surviving to independence (i.e., ≥3 months of age) were used to estimate components of male genetic RS at each age (age‐specific RS) and throughout life (lifetime RS or “LRS”): age‐specific and lifetime EGP (the number of young sired outside a male's social group) and WGP (the number of young sired within a male's social group). For each male, age‐specific and lifetime social (“apparent”) RS (RS_ap_) were estimated as the total number of young (per age or throughout life, respectively) who were produced by the male's social female(s) and not necessarily sired by the males (see Table 1 for a summary of the different paternity terms we use). Males not occupying a dominant position (at a given age or throughout life) were assigned RS_ap_ of zero (at that age or throughout life, respectively), because they were not socially bonded to female(s).

**Table 1:** Glossary of paternity terms

Term	Abbreviation	Description
Age‐specific		At age x
Lifetime		Throughout life
Extra‐pair paternity	EPP	Number of young sired outside the pair bond
Within‐pair paternity	WPP	Number of young sired within the pair bond
Extra‐group paternity	EGP	Number of young sired outside a male's social group
Within‐group paternity	WGP	Number of young sired within a male's social group
Reproductive success	RS	Number of (extra‐group + within‐group) young sired (i.e., genetic offspring)
Apparent reproductive success	RS_ap_	Number of young produced by the social female(s) of a male and sired by either the focal male or other males (i.e., social offspring)
Lifetime EGP	LEGP	Number of young sired outside a male's social group throughout the male's life
Lifetime WGP	LWGP	Number of young sired within a male's social group throughout the male's life
Lifetime reproductive success	LRS	Number of (extra‐group + within‐group) young sired (i.e., genetic offspring) throughout life
Lifetime apparent reproductive success	LRS_ap_	Number of young produced by the social female(s) of a male (i.e., social offspring) throughout the male's life and sired by either the focal male or other males

### VARIANCE PARTITIONING AND STATISTICAL ANALYSES

First, we assessed how much variance EGP contributed to LRS. We partitioned the variance in LRS into its (co)variance components and quantified the total contribution of lifetime EGP and WGP to the variance in LRS. LRS is the sum of lifetime EGP and WGP, so the variance in LRS, var(LRS), can be partitioned into the variances in lifetime EGP and WGP, var(lifetime EGP) and var(lifetime WGP), and their covariance, cov(lifetime EGP, lifetime WGP) (Webster et al. 1995):

(1) $$\begin{array}{ccc} \mathrm{var}\left(\mathrm{LRS}\right) & = & \mathrm{var}\left(\mathrm{lifetime}\;\mathrm{EGP}\right)+\mathrm{var}\left(\mathrm{lifetime}\;\mathrm{WGP}\right)\\ & & +\;2\mathrm{cov}\left(\mathrm{lifetime}\;\mathrm{EGP},\;\mathrm{lifetime}\;\mathrm{WGP}\right) \end{array}$$

**Figure 1:**
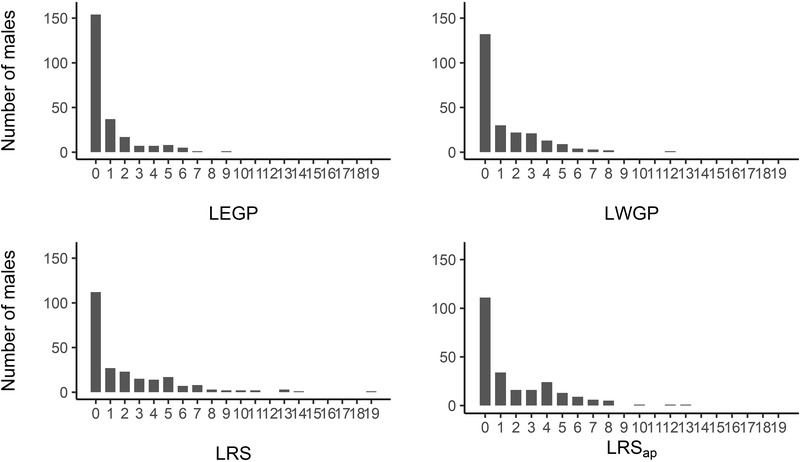
**Distribution of lifetime extra‐group paternity (LEGP, top left), within‐group paternity (LWGP, top right), reproductive success (LRS, bottom left), and apparent reproductive success (LRS_ap_, bottom right) in male Seychelles warblers (*n* = 237)**. Genetic paternity measures—LEGP, LWGP, and LRS—consist of the number of extra‐group, within‐group, and total offspring sired by males throughout life. The social (“apparent”) reproduction measure LRS_ap_ corresponds to the number of young produced by a male's social female(s), and not necessarily sired by that male, throughout the male's life

Second, we assessed at what ages the contribution of age‐specific EGP to the variance in LRS was highest. We quantified the contribution of age‐specific EGP and WGP to the variance in LRS across all males, accounting for their longevity. To do so, all variances of age‐specific RS, and all (co)variance components of age‐specific RS, are required to add up to the total variance in LRS, var(LRS). To fulfill this requirement, we employed the “additive method” of variance partitioning (Arnold and Wade 1984; Koenig et al. 1991), which involves assigning an age‐specific RS of zero to individuals who have died at earlier ages than the oldest observed individual(s). Thus, the variance in LRS, var(LRS), can be partitioned into:

(2) $$\mathrm{var}\left(\mathrm{LRS}\right)=\sum_{i=1}^{n}\mathrm{var}\left({\mathrm{RS}}_{i}\right)+\sum_{j>i\geq 1}^{n}2\mathrm{cov}\left({\mathrm{RS}}_{i},{\mathrm{RS}}_{j}\right)\;$$

where var(RS_i_) and var(RS_j_) are the variances in RS at ages i and j (age‐specific variances), respectively, cov(RS_i_, RS_j_) is the covariance between the RS at age i and j (between‐age covariance), and *n* is the maximum age considered (Arnold and Wade 1984; Koenig et al. 1991; Lebigre et al. 2013). Var(LRS) can be further partitioned into its age‐specific EGP and WGP (co)variance components:

(3) $$\begin{array}{ccc} & & \mathrm{var}\left(\mathrm{LRS}\right)=\sum_{i=1}^{n}\mathrm{var}\left(\mathrm{EG}P_{i}\right)+\sum_{i=1}^{n}\mathrm{var}\left(\mathrm{WG}P_{i}\right)\\ & & \;+\sum_{j>i\geq 1}^{n}2\mathrm{cov}\left(\mathrm{EG}P_{i},\mathrm{EG}P_{j}\right)+\sum_{j>i\geq 1}^{n}2\mathrm{cov}\left(\mathrm{WG}P_{i},\mathrm{WG}P_{j}\right)\\ & & \;+\sum_{i\geq 1}^{n}2\mathrm{cov}\left(\mathrm{EG}P_{i},\mathrm{WG}P_{i}\right)+\sum_{j>i\geq 1}^{n}2\mathrm{cov}\left(\mathrm{EG}P_{i},\mathrm{WG}P_{j}\right)\; \end{array}$$

where var(EGP_i_) and var(WGP_i_) are the age‐specific variances in EGP and WGP, respectively; cov(EGP_i_, EGP_j_) and cov(WGP_i_, WGP_j_) are the between‐age covariances in EGP and WGP; cov(EGP_i_, WGP_i_) and cov(EGP_i_, WGP_j_) are the age‐specific and between‐age covariances between EGP and WGP (Arnold and Wade 1984; Koenig et al. 1991; Lebigre et al. 2013). We estimated age‐specific (co)variances for 12 age classes. We grouped males aged ≥12 years into one class, as these were rare in our dataset (<0.1%, see Fig. 1). All other age classes consist of 1specific year of age each, i.e., 1‐ to 11‐year‐old males.

Third, we assessed at what ages the contribution of EGP to the variance in age‐specific RS was highest. To do so, we employed the “independent method” of variance decomposition (Koenig and Albano 1987; Koenig et al. 1991), which estimates (co)variance components of age‐specific RS independent of longevity, by assigning missing values (rather than zero age‐specific RS) to individuals who died before each age. Using this method, we quantified the contribution of EGP and WGP to the variance in age‐specific RS of males who survived to each age (i.e., we plugged (co)variances in age‐specific RS, EGP, and WGP into Eq. [ 1]).

Fourth, we compared the variance in RS to that in RS_ap_ to assess whether EGP increased the opportunity for selection over that arising from the monogamous social mating system. Comparisons were performed by calculating the ratio of RS over RS_ap_ at three levels: overall (i.e., lifetime measures), at each age across all males (i.e., age‐specific measures estimated with the additive method), and at each age across males surviving to that age (i.e., age‐specific measures estimated with the independent method).

All estimated (co)variances were standardized by dividing them by the squared mean of RS (or RS_ap_) to quantify the “opportunity for selection” and to allow comparison with other studies. Lifetime (co)variances as well as age‐specific (co)variance components of LRS or LRS_ap_ (estimated with the additive method) were standardized by the squared mean of LRS or LRS_ap_; (co)variances in the age‐specific RS or RS_ap_ of males surviving to each age (estimated with the independent method) were mean standardized within ages (i.e., divided by the corresponding age‐specific squared mean of RS or RS_ap_). For each standardized (co)variance value, we estimated the 95% confidence interval (CI), which we used to determine if values differed significantly from one another (two values were considered to vary significantly if their CIs did not overlap). The CIs we generated were bias‐corrected accelerated CIs, estimated using non‐parametric bootstrapping with the R package boot (1.3.24; Canty and Ripley 2020).

Finally, we assessed whether variance in male lifetime reproduction (total, extra‐group, and within‐group) was reflected by longevity and by the age of first dominance (first breeding opportunity), and whether variance in male LRS was reflected by the proportion of EGP gained in life (out of total LRS). For this, we built three generalized linear mixed models (GLMMs) with Poisson error structure (log link function) and birth year (“cohort”) fitted as random intercept. GLMM 1 regressed LRS (of males with LRS ≥ 1; *n =* 123) against three fixed predictors—the proportion of lifetime EGP, age of first dominance, and longevity. GLMMs 2 and 3 regressed lifetime EGP and WGP (of males who gained dominance in life; *n* = 182), respectively, against three fixed predictors: age of first dominance, longevity, and lifetime WGP (GLMM 2) or EGP (GLMM 3). Lifetime EGP and WGP were included in models 2 and 3, respectively, to assess the relationship between these two paternity measures. An observation‐level random intercept was included in GLMMs 2 and 3 to account for overdispersion (Harrison 2014). Models were fitted using the lme4 (1.1‐20) package (Bates et al. 2015) in R (3.6.3). We checked for collinearity between fixed effects using the variance inflation factor (VIF) and found none (VIF < 3). We standardized (mean‐centered and scaled to one standard deviation) continuous predictors and used the “BOBYQA” nonlinear optimization (Powell 2009) to aid convergence of models.

## Results

Throughout their lives, Seychelles warbler males born in 1997–2005 (*n* = 237) sired an average of 2.17 offspring that reached independence (range: 0–19, median: 1, mode: 0), of which 1.30 were within‐group offspring (range: 0–12, median: 0, mode: 0) and 0.87 were extra‐group offspring (range: 0–9, median: 0, mode: 0). Males raised an average of 1.88 social offspring, i.e., offspring produced by their social female(s) but not necessarily sired by them (range: 0–13, median: 1, mode: 0) (Fig. 1). Across these males, EGP gains accounted for *ca* 40% of mean LRS. Males who never gained a dominant position (*n* = 55) produced zero offspring (except for 2 males who produced 1 offspring each). Males who did gain dominance during their life (*n* = 182) had a mean age of first dominance of 1.8 years (range: 1–6, median: 2, mode: 2; Supporting information Fig. S1). The average lifespan across all males in our dataset was 5.1 years and the distribution of lifespan was heavily left‐skewed (lifespan range: 1–16, median: 4, mode: 1; see Supporting information Fig. S2). Nearly all (within‐ and extra‐group) young were sired by dominant males: only 2% (12/513) of offspring were sired by a subordinate male. The number of offspring that males sired in their life was left‐skewed. A total of 47% (112/237) of males produced no offspring at all and only 9% (22/237) of males sired more than six offspring (Fig. 1). A total of 65% (154/237) of males produced no extra‐group offspring and 56% (132/237) of males sired no within‐group offspring. Only, *ca* 1% (2/237) and 3% (6/237) of males produced more than six extra‐group and within‐group young, respectively (Fig. 1). The number of social offspring raised by males was also left‐skewed, with 47% (111/237) of males having no social offspring and only 6% (14/237) of males having more than six social offspring (Fig. 1).

### THE VARIANCE CONTRIBUTION OF LIFETIME EGP AND WGP TO LRS

**Figure 2:**
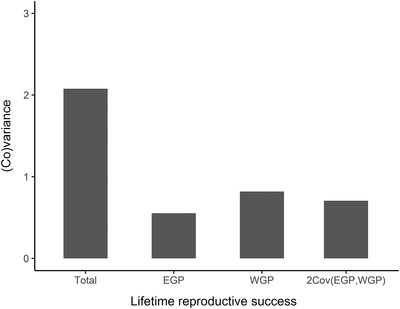
**The standardized variance in lifetime reproductive success and its lifetime (co)variance components for male Seychelles warblers (*n* = 237)**. The variance in lifetime reproductive success is partitioned into the variance in extra‐group paternity (EGP) and within‐group paternity (WGP), plus twice their covariance, 2Cov(EGP,WGP)

The standardized variance in LRS across male Seychelles warblers was 2.08 (Fig. 2, Table 2). The contributions of lifetime EGP and WGP to this variance were similar (with no statistically significant difference between the two), i.e., *ca* 27% and 39%, respectively. Twice the covariance between EGP and WGP, which was positive, accounted for 34% of the variance in LRS (Table 2, Supporting information Table S1).

**Table 2:** Additive method: standardized age‐specific (co)variance components of lifetime reproductive success of male Seychelles warblers (*n* = 237)

n = 237	EGP		WGP			RS		RS_ap_		EGP:WGP	RS:RS_ap_
Age (years)	Var	Mean	Var	Mean	2Cov(EGP,WGP)	Var	Mean	Var	Mean	Var ratio	Var ratio
1	0.002	0.008	0.006	0.03	−0.0001	0.008	0.04	0.02	0.07	0.29	0.39
	(0.00, 0.004)		(0.002, 0.01)		(−0.001, 0.00)	(0.004, 0.01)		(0.01, 0.04)			
2	0.02	0.10	0.03	0.16	0.006	0.06	0.25	0.07	0.24	0.65	0.86
	(0.01, 0.03)		(0.02, 0.04)		(−0.001, 0.02)	(0.04, 0.09)		(0.05, 0.09)			
3	0.03	0.09	0.05	0.23	0.004	0.08	0.32	0.10	0.33	0.57	0.80
	(0.02, 0.05)		(0.04, 0.07)		(−0.004, 0.02)	(0.06, 0.11)		(0.08, 0.17)			
4	0.03	0.12	0.04	0.16	0.003	0.07	0.27	0.09	0.26	0.76	0.84
	(0.02, 0.05)		(0.03, 0.07)		(−0.004, 0.01)	(0.05, 0.10)		(0.06, 0.12)			
5	0.04	0.14	0.03	0.12	0.006	0.07	0.25	0.07	0.20	1.34	1.09
	(0.02, 0.10)		(0.02, 0.05)		(−0.002, 0.02)	(0.05, 0.12)		(0.05, 0.10)			
6	0.04	0.12	0.03	0.14	0.02	0.09	0.27	0.07	0.21	1.37	1.33
	(0.02, 0.08)		(0.02, 0.04)		(0.005, 0.04)	(0.06, 0.15)		(0.05, 0.09)			
7	0.02	0.08	0.04	0.15	0.03	0.10	0.22	0.07	0.18	0.54	1.34
	(0.01, 0.05)		(0.03, 0.07)		(0.01, 0.10)	(0.05, 0.22)		(0.05, 0.11)			
8	0.02	0.06	0.02	0.08	0.007	0.04	0.14	0.04	0.11	0.89	0.98
	(0.008, 0.03)		(0.01, 0.03)		(0.001, 0.02)	(0.03, 0.07)		(0.02, 0.08)			
9	0.01	0.05	0.02	0.08	0.01	0.05	0.13	0.03	0.09	0.72	1.73
	(0.006, 0.03)		(0.01, 0.03)		(0.004, 0.04)	(0.02, 0.10)		(0.02, 0.05)			
10	0.009	0.05	0.02	0.07	0.01	0.04	0.11	0.04	0.09	0.50	0.98
	(0.004, 0.02)		(0.009, 0.05)		(0.003, 0.03)	(0.02, 0.09)		(0.02, 0.08)			
11	0.01	0.04	0.01	0.03	0.009	0.03	0.07	0.02	0.04	0.99	1.58
	(0.004, 0.03)		(0.002, 0.04)		(0.002, 0.03)	(0.01, 0.09)		(0.006, 0.06)			
12‐16	0.005	0.03	0.02	0.06	0.01	0.04	0.08	0.03	0.08	0.23	1.17
	(0.002, 0.01)		(0.01, 0.05)		(0.003, 0.03)	(0.02, 0.10)		(0.01, 0.07)			
Σ Var_(age‐specific)_ or Σ2Cov_(age‐specific)_	0.24		0.32		0.12	0.68		0.65		0.75	1.05
Σ2Cov_(between‐age)_	0.31		0.50		0.58	1.39		1.10			
**Lifetime**	**0.55**	**0.87**	**0.82**	**1.30**	**0.71**	**2.08**	**2.17**	**1.75**	**1.88**	**0.67**	**1.19**
	**(0.40, 0.78)**		**(0.63, 1.22)**		**(0.44, 1.15)**	**(1.54, 3.04)**		**(1.39, 2.38)**			

The variance in lifetime genetic reproductive success (RS) was partitioned into its age‐specific additive components. Variances (Var) in age‐specific RS and its components, i.e., age‐specific extra‐group paternity (EGP), within‐group paternity (WGP), and twice the covariances (2Cov) between EGP and WGP, add up to their respective sums of variances or doubled covariances “ΣVar_(age‐specific)_ or Σ2Cov_(age‐specific)_.” Twice the between‐age covariances in RS, EGP, and WGP, and between EGP and WGP, add up to their respective sums of covariances “Σ2Cov_(between‐age)_.” The sum of the ΣVar_(age‐specific)_ or Σ2Cov_(age‐specific)_ plus the Σ2Cov_(between‐age)_ gives the variances, or doubled covariances, in lifetime reproduction measures, found in the bottom row (“Lifetime”). The relative contribution of age‐specific EGP and WGP to the variance in age‐specific and lifetime RS is shown as the ratio of Var(EGP) over Var(WGP). The age‐specific (co)variance components of lifetime social (“apparent”) reproductive success (RS_ap_) and the variance ratio of overall and age‐specific RS over RS_ap_ are also shown. All (co)variances were standardized by the squared mean of lifetime RS (for genetic paternity measures: EGP, WGP, and RS) or lifetime RS_ap_ (for social reproduction). (Co)variances are associated with their 95% confidence interval (in brackets). For presentation purposes, values >0.01 were rounded up to two decimal places and values <0.01 were rounded up to the number of decimal places with a minimum of one significant figure.

### THE VARIANCE CONTRIBUTION OF AGE‐SPECIFIC EGP AND WGP TO LRS (ADDITIVE METHOD)

The additive method allowed us to partition the variance in LRS into its additive age‐specific (co)variance components across all males (by assigning an age‐specific RS of zero to individuals who died at earlier ages than the oldest observed individuals). This revealed that age‐specific contributions of EGP, WGP, and RS to the variance in LRS varied across ages and were all lowest in males aged 1 year. In particular, the variance contribution of age‐specific EGP to the variance in LRS was lowest at age 1, higher at ages 2–9, and again low at ages ≥10 (Table 2, Supporting information Fig. S3). The contribution of age‐specific WGP to the variance in LRS was also lowest at 1 year of age and significantly higher at ages 2–7, and again low at ages ≥8 (Table 2, Supporting information Fig. S3). Across ages, there were no statistically significant differences between the age‐specific relative contributions of WGP and EGP to the variance in LRS (Table 2). The variance contribution of age‐specific RS to total LRS was lowest at age 1 and higher at most ages ≥2 (i.e., ages 2–10 and ≥12; Table 2, Supporting information Fig. S3).

The age‐specific covariance between EGP and WGP was positive at all ages, except at the age of 1, when it was negative but with a near‐zero absolute value (|<0.001|; Table 2). Single covariance values, i.e., age‐specific (within‐age) covariances between EGP and WGP and between‐age EGP‐EGP, WGP‐WGP and EGP‐WGP covariances, were generally small, but the sum of age‐specific and between‐age covariances contributed, respectively, 6 and 28% of the total variance in LRS (Table 2, Supporting information Table S1).

### THE VARIANCE CONTRIBUTION OF AGE‐SPECIFIC EGP AND WGP TO AGE‐SPECIFIC RS (INDEPENDENT METHOD)

The independent method enabled us to partition the variance in age‐specific RS into its age‐specific (co)variance components across males surviving to each age (by assigning missing values to individuals that died before each age). This showed that, across different male age groups, the contribution of EGP to the variance in age‐specific RS was substantial and roughly as important as that of WGP. The standardized variance in age‐specific EGP and WGP (and, therefore, RS) of males surviving to each age was highest in males of 1 year (Table 3), likely due to the very low mean in EGP (0.008) and WGP (0.03) in this age class (Table 3). However, due to its very wide CI, the standardized variance in age‐specific EGP did not differ significantly from other age‐specific variance values, which, from age 2 onward, did not vary substantially across males of different ages (Table 3). The standardized variance in age‐specific WGP, as well as RS, was significantly higher at age 1 and lower thereafter (Table 3). The relative contributions of age‐specific EGP and WGP to the variance in age‐specific RS did not differ significantly from one another across ages, except in males ≥12 years old, where WGP contributed more than EGP. 75% of the age‐specific covariances between EGP and WGP were positive; absolute values of covariances were usually smaller than those of variances of both EGP and WGP (significantly so at ages 3–6; Table 3).

**Table 3:** Independent method: standardized (co)variance components of the age‐specific reproductive success of male Seychelles warblers (*n* = 237) that survive to each age

		EGP		WGP			RS		RS_ap_		EGP:WGP	RS:RS_ap_
Age (years)	N	Var	Mean	Var	Mean	2Cov(EGP,WGP)	Var	Mean	Var	Mean	Var ratio	Var ratio
1	237	5.83	0.008	19.96	0.03	−0.35	25.44	0.04	15.73	0.07	0.29	1.62
		(0.00, 14.38)		(5.83, 33.47)		(−1.90, 0.00)	(11.55, 41.29)		(8.90, 27.82)			
2	193	1.20	0.12	1.83	0.19	0.28	3.31	0.31	3.33	0.29	0.66	0.99
		(0.79, 1.86)		(1.36, 2.56)		(−0.14, 1.08)	(2.42, 5.08)		(2.54, 4.40)			
3	162	0.88	0.14	1.41	0.33	−0.02	2.27	0.47	1.95	0.49	0.62	1.17
		(0.47, 1.47)		(1.09, 1.85)		(−0.29, 0.31)	(1.74, 3.05)		(1.49, 3.17)			
4	134	0.96	0.21	1.24	0.28	−0.11	2.09	0.49	2.15	0.46	0.78	0.97
		(0.63, 1.45)		(0.84, 2.14)		(−0.39, 0.23)	(1.58, 2.94)		(1.64, 2.96)			
5	108	1.17	0.30	0.87	0.26	−0.08	1.96	0.56	2.30	0.44	1.34	0.85
		(0.66, 2.83)		(0.59, 1.31)		(−0.38, 0.28)	(1.34, 3.23)		(1.65, 3.41)			
6	85	0.85	0.34	0.53	0.40	0.06	1.44	0.74	1.32	0.58	1.60	1.09
		(0.49, 1.46)		(0.42, 0.76)		(−0.19, 0.40)	(0.95, 2.41)		(1.04, 1.69)			
7	67	0.51	0.27	0.79	0.52	0.56	1.87	0.79	1.61	0.63	0.65	1.16
		(0.27, 1.18)		(0.55, 1.32)		(0.11, 1.85)	(1.01, 4.38)		(1.18, 2.32)			
8	56	0.86	0.25	0.87	0.34	0.03	1.76	0.59	2.36	0.46	1.00	0.75
		(0.43, 1.56)		(0.58, 1.36)		(−0.35, 0.48)	(1.24, 2.79)		(1.48, 4.15)			
9	42	0.56	0.29	0.64	0.45	0.32	1.53	0.74	1.41	0.50	0.87	1.08
		(0.29, 1.01)		(0.44, 1.01)		(−0.04, 1.17)	(0.87, 3.27)		(1.16, 2.49)			
10	34	0.36	0.32	0.70	0.47	0.18	1.23	0.79	1.58	0.65	0.51	0.78
		(0.20, 0.41)		(0.37, 2.06)		(−0.12, 0.72)	(0.65, 3.23)		(1.00, 2.86)			
11	30	1.25	0.30	1.38	0.23	0.70	3.33	0.53	5.48	0.30	0.91	0.61
		(0.51, 2.36)		(0.33, 4.53)		(0.00, 2.47)	(1.49, 8.27)		(1.60, 14.93)			
12‐16	22	0.25	0.27	0.98	0.64	0.37	1.60	0.91	1.09	0.82	0.26	1.48
		(0.10, 0.31)		(0.54, 1.90)		(−0.08, 0.99)	(0.68,3.18)		(0.65,2.13)			
**Lifetime**	**237**	**0.55**	**0.87**	**0.82**	**1.30**	**0.71**	**2.08**	**2.17**	**1.75**	**1.88**	**0.67**	**1.19**
		**(0.40, 0.78)**		**(0.63, 1.22)**		**(0.44, 1.15)**	**(1.54, 3.04)**		**(1.39, 2.38)**			

Variances (Var) in age‐specific genetic reproductive success (RS) of males surviving to each age were partitioned into their within‐ and extra‐group paternity components. At each age, the variance in age‐specific RS and its components i.e., age‐specific extra‐group paternity (EGP), within‐group paternity (WGP), and twice the covariances (2Cov) between EGP and WGP, are associated to the number of males alive at that age (N). The relative contribution of EGP and WGP to the variance in age‐specific RS is shown as the ratio of Var(EGP) over Var(WGP). The age‐specific variances in social (“apparent”) reproduction (RS_ap_) and the variance ratio of RS over RS_ap_ are also shown. Age‐specific (co)variances were standardized by the squared mean of the corresponding age‐specific RS (for genetic paternity measures: EGP, WGP, and RS) or RS_ap_ (for social reproduction). (Co)variances are associated with their 95% confidence interval (in brackets). Lifetime measures are shown in the bottom row for comparison. For presentation purposes, values >0.01 were rounded up to two decimal places and values <0.01 were rounded up to the number of decimal places with a minimum of one significant figure.

### RS *VS* RS_AP_ (ADDITIVE AND INDEPENDENT METHODS)

The additive and independent methods showed that there was no statistically significant difference between the variation in lifetime or age‐specific RS *vs* RS_ap_. In particular, the standardized variance in LRS was 2.08 while that in LRS_ap_ was 1.75, but the two did not differ significantly from one another (Table 2). The variance ratio of LRS to LRS_ap_ was 1.19, indicating that EGP increased the variance in LRS by 19% over the variance arising from the social mating system, though this increment was not statistically significant (Table 2). Partitioning the variance in LRS_ap_ with the additive method revealed that the contribution of variances in age‐specific RS_ap_ was lowest at age 1, significantly higher from 2 years onward, and low again at ages ≥8 (Tables 1,Supporting information Table S1, Fig. S4). The age‐specific variance ratio of RS over RS_ap_ was ≥0.80 across all ages, except for age 1, when the ratio was 0.39, but age‐specific variances in RS and RS_ap_ did not differ from one another in a statistically significant way at any age (Table 2).

When estimating variances with the independent method, the standardized variance in age‐dependent RS_ap_ was highest in males who survived to 1 year of age and was lower in males ≥2 years old (Table 3). The variance ratio of age‐specific RS to RS_ap_ had values between 1 and 1.6 across over half of the male age groups (Table 3), suggesting that the variance in age‐specific RS was higher than the variance in age‐dependent RS_ap_ across the majority of age groups, but differences between RS and RS_ap_ were not statistically significant.

### DRIVERS OF VARIATION IN LIFETIME REPRODUCTION

Among successful breeders (i.e., males siring at least one offspring), the proportion of EGP gained in life (out of total lifetime reproduction) had a positive effect on LRS (Table 4). Not surprisingly, longevity had a large positive effect on LRS, while a later age of first dominance (i.e., breeding opportunity) had a negative effect (Table 4). Longevity was also positively associated with both lifetime EGP and lifetime WGP acquired by males who gained dominance in their life (Table 5). Lifetime WGP, but not lifetime EGP, was negatively related to age of first dominance (Table 5). After controlling for age of first dominance and longevity, lifetime EGP and WGP were not significantly related to one another in males who gained a breeding spot in life (Table 5).

**Table 4:** Generalized linear mixed model (GLMM) of lifetime reproductive success of male Seychelles warblers that gain dominance and sire ≥1 offspring during their life, in relation to longevity, age of first dominance, and the proportion of extra‐group offspring sired in life out of all offspring sired in life (*n* = 123 males)

	**Lifetime Reproductive Success**		
**Fixed term**	***β***	***SE***	***p***
Intercept	1.29	0.07	<0.001
AFD	**−0.15**	**0.05**	**0.003**
Longevity	**0.50**	**0.04**	**<0.001**
Proportion lifetime EGP	**0.10**	**0.05**	**0.045**
**Random term**	***σ^2^***	***n***	
Cohort	0.02	9	

Coefficient estimates (*β*), standard errors (*SE*), and *p* values (*p*) are shown for each fixed effect.

AFD = age of first dominance, Proportion lifetime EGP = proportion of extra‐group offspring sired in life.

Variance (σ^2^) and number of observations (*n*) are shown for each random effect. The GLMM was built with a Poisson error structure. Significant predictors (*p *< 0.05) are shown in bold.

**Table 5:** Generalized linear mixed models (GLMMs) of lifetime extra‐group paternity (EGP) and within‐group paternity (WGP) gained by male Seychelles warblers that obtain a dominant position during their life, in relation to longevity, age of first dominance, and either lifetime WGP or EGP, respectively (*n* = 182 males)

	**Lifetime EGP**			**Lifetime WGP**		
**Fixed term**	***β***	***SE***	***p***	***β***	***SE***	***p***
Intercept	−0.56	0.15	<0.001	−0.06	0.12	0.599
AFD	−0.20	0.11	0.073	−**0.17**	**0.07**	**0.018**
Longevity	**0.76**	**0.14**	**<0.001**	**0.85**	**0.09**	**<0.001**
Lifetime WGP	0.09	0.13	0.462	‐	‐	‐
Lifetime EGP	‐	‐	‐	0.01	0.06	0.918
**Random term**	***σ^2^***	***n***		***σ^2^***	***n***	
Cohort	0.00	9		0.04	9	
Male ID	0.69	182		0.15	182	

Coefficient estimates (*β*), standard errors (*SE*), and *p* values (*p*) are shown for each fixed effect.

AFD = age of first dominance. Variance (σ^2^) and number of observations (*n*) are shown for each random effect. The GLMMs were built with a Poisson error structure. Observation identity was added as a random effect to to eliminate overdispersion. Significant predictors (*p *< 0.05) are shown in bold.

## Discussion

### THE TOTAL OPPORTUNITY FOR SELECTION VIA LIFETIME EGP

In male Seychelles warblers, the contributions of EGP (27%) and WGP (39%) to the variance in LRS were similar, indicating that both EGP and WGP provide an important contribution to the total opportunity for selection. The positive covariance between lifetime EGP and WGP (accounting for 34% of the total variance in LRS) indicates that Seychelles warblers that sire more within‐group offspring also sire more extra‐group young. These results are comparable to those found by the, to our knowledge, only other study that has assessed the contribution of infidelity to the variance in male LRS in a socially monogamous cooperative breeder, the splendid fairy‐wren (Webster et al. 2007). On the other hand, the few other studies that have analyzed near‐complete reproductive data to decompose the variance in lifetime reproduction in socially monogamous populations, found EPP to contribute little to the total opportunity for selection compared to WPP, i.e., 18 *vs* 45% in song sparrows (Lebigre et al. 2012), 4 *vs* 88% in white‐throated sparrows (Grunst et al. 2019).

The strong positive effect of longevity on both lifetime EGP and WGP (and, hence, also LRS) of male Seychelles warblers, revealed by our models, indicates that the positive covariance between EGP and WGP is due to longevity. That is because individuals with longer lifespans can engage in more reproductive events and, therefore, obtain higher LRS, as has been shown in many species (e.g., Clutton‐Brock 1988; Merilä and Sheldon 2000; but see Herényi et al. 2012). Our models showed that lifetime EGP and WGP were not significantly related, which seems in apparent contrast with the positive EGP‐WGP covariance we found in the male population. However, the lack of a relationship between EGP and WGP in our models resulted from controlling for the effect of longevity (and age of first dominance) and only considering males who gained dominance (i.e., a breeding spot) in life. Across the whole male population, Seychelles warblers that acquire more lifetime EGP also gain more lifetime WGP, and this effect is driven by longevity.

Longer‐lived males (and individuals gaining dominance earlier in life) may also have the opportunity to acquire a higher number of mates in life, and variation in this number may increase the opportunity for selection (Webster et al. 1995; Germain et al. 2021). In fact, in Seychelles warblers, a substantial amount of the variance in male LRS results from the variation in the number of mates that a male has in life, i.e., 26 and 32% for extra‐ and within‐group mates, respectively (while 34% results from the positive covariance between them, indicating that males with more within‐group mates also have more extra‐group mates; Supporting information Table S2). Interestingly, variation in the number of within‐ and extra‐group mates contributes a similar amount of variance to LRS. In particular, variation in the number of within‐group mates may derive from a combination of different sources, including the number of within‐group sexually mature female subordinates whom a male can copulate with, the number of territories a male has dominance in (rarely, but occasionally, >1), and the amount of stochastic turnover in social mates due to mortality.

Comparing LRS and LRS_ap_ showed that lifetime EGP increased the total opportunity for selection in males (over that arising from the monogamous mating system) by 19%, but this increase was not statistically significant. A total of 19% is a considerably lower value than the increase in variance found in most earlier studies which only analyzed annual male reproduction (often >200%, range: 3–1330%; see references in Lebigre et al. 2012). It is, however, comparable to that found in the few other studies that examined male lifetime reproduction (range: 1–20%; Webster et al. 2007; Lebigre et al. 2012; Grunst et al. 2019).

### THE TOTAL OPPORTUNITY FOR SELECTION VIA AGE‐SPECIFIC EGP (ADDITIVE METHOD)

Among all males, the age‐specific contribution of EGP to the total opportunity for selection ranged from 0.09 to 1.98% across ages, and was similar to the contribution of WGP (range: 0.30−2.43%), indicative of a similarly important age‐specific contribution of EGP and WGP to the total opportunity for selection. On the other hand, the other study (Lebigre et al. 2013) that assessed the age‐specific components of the variance in LRS (and LRS_ap_) showed that, in song sparrows, the age‐specific contribution of WGP was higher than that of EGP across ages.

In Seychelles warblers, males increase both their within‐ and extra‐group offspring production till *ca* 6 and 8 years, respectively, after which they show a senescent decline (Raj Pant et al. 2020). In particular, at the age of 1 year, most males are unable to successfully breed and those who attempt to do so in their territory are most likely to be cuckolded (Raj Pant et al. 2020). Here, we found that the age‐specific contributions of EGP and WGP to the total opportunity for selection were lowest at age 1, as almost no offspring were sired by 1‐year‐old males, including *ca* 55% of males who had gained dominance at the age of 1. The age‐specific variance contributions of both EGP and WGP increased significantly from 1‐ to 2‐year old males. This is likely because of an age‐related increase in the ability of males to guard paternity and sire within‐ and extra‐group offspring, with an evident improvement occurring from the age of 1 (when males attempt to reproduce for the first time) to the age of 2 years. This improvement in obtaining paternity is possibly mediated by an age‐dependent increase in breeding experience and/or physiological changes in early life, resulting, for instance, in improvements in body condition, ejaculate competitiveness, timing of copulations, mate‐guarding ability, and effectiveness in finding and copulating with fertile extra‐pair females (Westneat and Stewart 2003; Hsu et al. 2015; Nakagawa et al. 2015).

Comparing the variance contributions of age‐specific RS *vs* RS_ap_ (to the variance in LRS and LRS_ap_, respectively) showed that the EGP‐mediated increases of the former over the latter (by 9–73%, in about half of the ages), were not statistically significant. Our results differ from those of Lebigre et al. (2013), who found that, in song sparrows, age‐specific EPP did significantly increase the variance contribution of age‐specific RS to LRS, beyond that arising from monogamy, at the youngest and oldest ages, and did so to a more variable extent (4−251%) across ages.

### THE AGE‐SPECIFIC OPPORTUNITY FOR SELECTION VIA AGE‐SPECIFIC EGP (INDEPENDENT METHOD)

Among males surviving to each age, the variance contributed by age‐specific EGP to the age‐specific RS was substantial (23–59% across ages) and not dissimilar from that of WGP (37–78%). The age‐specific opportunity for selection was highest in 1‐year‐old males and lower from 2 years of age, a result which is consistent with the one other study that assessed this (Lebigre et al. 2013) and with the pattern of early‐life improvement in paternity acquisition and guarding that occurs in male Seychelles warblers (especially from 1 to 2 years of age). On the other hand, in contrast with song sparrows, where EGP significantly increased the age‐specific variances in RS over RS_ap_ (Lebigre et al. 2013), in Seychelles warblers, the EGP‐mediated increases in the opportunity for selection, which we found at most ages (by 8–62%), were not statistically significant.

### IMPLICATIONS AND FUTURE DIRECTIONS

EPP has been widely hypothesized to be a key mechanism underlying sexual selection in socially monogamous species, many of which feature sexually dimorphic traits, despite the low (apparent) variation in mating success (Andersson 1994; Webster et al. 1995; Grunst et al. 2019). The Seychelles warbler is a good candidate species to test this prediction, as it displays a socially monogamous and genetically promiscuous breeding system, as well as sexual dimorphism in body size (males being larger than females; Kingma et al. 2016b) and song (males singing more frequently and more complex songs than females; Catchopole and Komdeur 1993). However, we found that, despite contributing substantially to the opportunity for selection (to a similar extent as WGP), EGP does not cause the variance in lifetime and age‐specific RS to be significantly higher than the variance arising from the social mating system (RS_ap_). Therefore, EGP is likely to play a role in shaping sexual selection in the Seychelles warbler, despite probably not being the main mechanism via which sexual selection can act in this species.

Other mechanisms through which sexual selection is predicted to act in socially monogamous species are a male‐biased adult sex‐ratio, causing some males to obtain mates while others do not (Price 1984; Dearborn et al. 2001), and variance in the quality of social mates that males manage to attract, with higher‐quality females producing more offspring (Kirkpatrick et al. 1990; Jones and Ratterman 2009). In the Seychelles warbler population on Cousin, sexual selection cannot act via male‐biased sex ratio, as the adult sex ratio is on average slightly, but significantly, female‐biased (mean ± SD = 0.48 ± 0.03; F. J. D. Speelman, unpubl. data).

Furthermore, social mate choice is also unlikely to drive sexual selection, because the combination of habitat saturation, social fidelity, and longevity are thought to constrain the choice of social mates in the population (Richardson et al. 2005; Wright et al. 2015). Since 1982, the Seychelles warbler population on Cousin has been at a carrying capacity of *ca* 320 birds residing in 110 territories, causing a surplus of unpaired adult birds without an independent breeding position (Komdeur 1992; Komdeur et al. 1995, Komdeur et al. 2016). This shortage of breeding spots is accentuated by the fact that Seychelles warblers generally form pairs for life and such pairs can last for many years due to long individual lifespan. Therefore, Seychelles warblers will likely occupy a breeding spot as soon as this becomes available, without much of an opportunity to actively choose between different social mates. However, variation in mate breeding quality, independent of social mate choice, may still affect variation in the reproductive output of males. In fact, about 38% of the variance in LRS (of males gaining EGP and WGP in life) derives from variation in the fecundity of within‐group mates, less so (14%) from variation in extra‐group female fecundity (Supporting information Table S2). This suggests that both social and, to a lower extent, extra‐group mate breeding quality, contribute to the opportunity for selection in Seychelles warblers.

Another potential mechanism via which sexual selection may act is habitat saturation itself. In fact, in the Seychelles warbler, the pressure to obtain a territory and occupy a dominant position is stronger in males than females (Eikenaar et al. 2009) because subordinate males almost never breed, while subordinate females do reproduce (Richardson et al. 2001; Hadfield et al. 2006; Hammers et al. 2019, Raj Pant et al. 2019). It is possible that habitat saturation plays a greater role in the variation in male RS compared to EGP, though the two mechanisms do not exclude one another and may act in concert. For instance, habitat saturation may increase the density of territories and individuals in an area, thus, increasing the opportunity, for males who gain dominance in that area, to obtain more EGP. In the Seychelles warbler population on Cousin, habitat saturation has increased territorial and individual density (Komdeur et al. 1995, Komdeur et al. 2016). Moreover, local dominant male density is used by male Seychelles warblers as a cue to assess paternity risk, with males adjusting their mate‐guarding rate accordingly (Komdeur 2001).

Overall, in our study, even though comparisons of the variance in RS *vs* RS_ap_ showed that EGP did not provide a statistically significant increase in the former over the latter, variance decomposition analyses revealed that EGP provided a considerable contribution to the total and age‐specific opportunities for selection (to a similar extent as WGP). In addition to simple comparisons of RS and RS_ap_, it may therefore, be helpful to address the issue with other analyses, such as the estimation of lifetime and age‐specific Bateman gradients, which explicitly quantify the opportunity for sexual selection (see Webster et al. 2007), though this was beyond the scope of our study. In addition, given that the opportunity for selection is a measure of the maximum possible strength of selection, rather than actual force of selection on a particular trait, selection gradient analyses are recommended to assess the strength of (sexual) selection via EGP on traits of interest such as body size and song structure. Moreover, further research is required to shed more light on the factors driving the variance in EGP, including investigations on the conditions that may promote male EGP, such as the number of social females and helpers in a male's group, the number of years since a pair‐bond between a male and his social female was formed, and whether a male's social mate participates in extra‐group mating or not. Finally, further work is required to investigate the variation in the RS of females and the ratio of this variation to the variation in male reproductive output.

## CONCLUSIONS

In the Seychelles warbler, the overall variance contribution of lifetime EGP to the total opportunity for selection was substantial, yet it did not significantly increase the variance in RS over that arising from the apparent (social) mating system. This indicates that, in this species, EGP constitutes a mechanism via which sexual selection can act, despite probably not being the main mechanism. This finding is in contrast with many past studies that found EPP to greatly increase the opportunity for selection; it highlights the importance of complete (or at least near‐complete) sampling of males over their lifetimes and of implementing statistical tests when assessing the effect of EPP on the opportunity for selection. Partitioning the total opportunity for selection (across all males) and the age‐specific opportunity for selection (of males surviving to each age) into their age‐specific (co)variance components, revealed that the contribution of EGP was as important as that of WGP, and varied considerably between 1‐year‐old and older males. Therefore, despite probably not playing the major role in shaping sexual selection, EGP provided a substantial contribution to the lifetime and age‐specific opportunity for selection, potentially influencing evolutionary processes in a wild population. Further research is now required for a deeper understanding of what factors drive the variance in EGP itself, and to quantify and compare the effect of habitat saturation *vs* EGP on the opportunity for selection.

## AUTHOR CONTRIBUTIONS

SRP and JK designed the study. SRP, MH and DSR contributed to fieldwork and HLD performed parentage assignment. SRP performed analyses, which MAV contributed to, and wrote the manuscript. All authors discussed the results and contributed to the manuscript. JK, DSR, HD, and TB manage the long‐term Seychelles warbler project.

## DATA ARCHIVING

Data has been archived in the Dryad Digital Repository (https://doi.org/10.5061/dryad.dfn2z350d).

## CONFLICT OF INTEREST

The authors declare no conflict of interest.

Associate Editor: Dr. Matthew D Dean

Handling Editor: Dr. Andrew G McAdam

## Supporting information

Figure S1. Distribution of ages at first dominance (years) among Seychelles warbler males who gained a dominant (breeding) position during their life (*n* = 182).
Figure S2. Distribution of lifespan (years) among Seychelles warbler males (*n* = 237).
Figure S3. Additive method: standardized age‐specific (co)variance components of the variance in lifetime reproductive success of male Seychelles warblers (*n* = 237).
Figure S4. Additive method: standardized age‐specific (co)variance components of the variance in the lifetime reproductive success – genetic (A) and social (B) – of male Seychelles warblers (*n* = 237).
Table S1. Additive method: percentage contribution of standardized age‐specific (co)variance components to lifetime reproductive success of male Seychelles warblers (*n* = 237).
Table S2. Decomposition of the variance in lifetime reproductive success (LRS) of male Seychelles warblers that obtained at least one within‐group (WG) and extra‐group (EG) offspring in life (*n* = 74) into the variance in lifetime within‐ and extra‐group: number of mates (M), female fecundity (number of offspring per mate, N) and paternity allocation (proportion of young sired per mate, P), following Webster et al. (1995).

Supplementary information

Supplementary information

Supplementary information

Supplementary information

## References

[evo14473-bib-0001] Ackay, E. , and J. Roughgarden . 2007. Extra‐pair reproductive activity in birds: review of the genetic benefits. Evolutionary Ecology Research 9:855–868.

[evo14473-bib-0002] Albrecht, T. , J. Schnitzer , J. Kreisinger , A. Exnerova , J. Bryja , and P. Munclinger . 2007. Extrapair paternity and the opportunity for sexual selection in long‐distant migratory passerines. Behavioral Ecology 18:477–486.

[evo14473-bib-0003] Andersson, M. 1994. Sexual selection. Princeton, NJ: Princeton University Press.

[evo14473-bib-0004] Arnold, S. J. , and M. J. Wade . 1984. On the measurement of natural and sexual selection: theory. Evolution; International Journal of Organic Evolution 38:709–719. 28555816 10.1111/j.1558-5646.1984.tb00344.x

[evo14473-bib-0005] Bates, D. , M. Mächler , B. Bolker , and S. Walker . 2015. Fitting linear mixed‐effects models using lme4. Journal of Statistical Software 67:1–48.

[evo14473-bib-0006] Bebbington, K. , S. A. Kingma , E. A. Fairfield , H. L. Dugdale , J. Komdeur , L. G. Spurgin , D. S. Richardson , and J. E. Strassmann . 2017. Kinship and familiarity mitigate costs of social conflict between Seychelles warbler neighbors. Proceedings of the National Academy of Sciences 114:E9036–E9045. 10.1073/pnas.1704350114 PMC5664493 29073100

[evo14473-bib-0007] Brommer, J. E. , J. Merila , and H. Kokko . 2002. Reproductive timing and individual fitness. Ecology Letters 5:802–810.

[evo14473-bib-0008] Brouwer, L. , I. Barr , M. Van De Pol , T. Burke , J. Komdeur , and D. S. Richardson . 2010. MHC‐dependent survival in a wild population: evidence for hidden genetic benefits gained through extra‐pair fertilizations. Molecular Ecology 19:3444–3455. 20670363 10.1111/j.1365-294X.2010.04750.x

[evo14473-bib-0009] Brouwer, L. , and S. C. Griffith . 2019. Extra‐pair paternity in birds. Molecular Ecology 28:4864–4882. 31587397 10.1111/mec.15259 PMC6899757

[evo14473-bib-0010] Canty, A. , and B. Ripley . 2020. boot: Bootstrap R (S‐Plus) Functions. R package version 1.3‐25.

[evo14473-bib-0011] Catchopole, C. K. , and J. Komdeur . 1993. The song of the Seychelles Warbler *Acrocephalus sechellensis*, an island endemic. Ibis 135:190–195.

[evo14473-bib-0012] Cleasby, I. R. , and S. Nakagawa . 2012. The influence of male age on within‐pair and extra‐pair paternity in passerines. Ibis 154:318–324.

[evo14473-bib-0013] Clutton‐Brock, T. H. 1988. Reproductive success. Studies of individual variation in contrasting breeding systems. Chicago, IL: University of Chicago Press.

[evo14473-bib-0014] Collet, J. , D. S. Richardson , K. Worley , and T. Pizzari . 2012. Sexual selection and the differential effect of polyandry. Proceedings of the National Academy of Sciences of the United States of America 109:8641–8645. 22592795 10.1073/pnas.1200219109 PMC3365207

[evo14473-bib-0015] Coulson, T. , and S. Tuljapurkar . 2008. The dynamics of a quantitative trait in an age‐structured population living in a variable environment. American National 172:599–612. 10.1086/591693 PMC3345271 18840061

[evo14473-bib-0016] Dearborn, D. C. , A. D. Anders , and P. G. Parker . 2001. Sexual dimorphism, extrapair fertilizations, and operational sex ratio in great frigatebirds (*Fregata minor*). Behavioral Ecology 12:746–752.

[evo14473-bib-0017] Dillard, J. R. 2017. High rates of extra‐pair paternity in a socially monogamous beetle with biparental care. Ecological Entomology 42:1–10.

[evo14473-bib-0018] Dolan, A. C. , M. T. Murphy , L. J. Redmond , K. Sexton , and D. Duffield . 2007. Extrapair paternity and the opportunity for sexual selection in a socially monogamous passerine. Behavioral Ecology 18:985–993.

[evo14473-bib-0019] Edwards, H. A. , H. L. Dugdale , D. S. Richardson , J. Komdeur , and T. Burke . 2018. Extra‐pair parentage and personality in a cooperatively breeding bird. Behavioral Ecology and Sociobiology 72:37. 29491549 10.1007/s00265-018-2448-z PMC5814466

[evo14473-bib-0020] Eikenaar, C. , D. S. Richardson , L. Brouwer , R. Bristol , and J. Komdeur . 2009. Experimental evaluation of sex differences in territory acquisition in a cooperatively breeding bird. Behavioral Ecology 20:207–214.

[evo14473-bib-0021] Engen, S. , R. Lande , and B.‐E. Saether . 2005a. Effective size of a fluctuating age‐structured population. Genetics 170:941–954. 15834152 10.1534/genetics.104.028233 PMC1450432

[evo14473-bib-0022] Engen, S. , R. Lande , B.‐E. Saether , and H. Weimerskirch . 2005b. Extinction in relation to demographic and environmental stochasticity in age‐structured models. Mathematical Biosciences 195:210–227. 15907948 10.1016/j.mbs.2005.02.003

[evo14473-bib-0023] Freeman‐Gallant, C. R. , N. T. Wheelwaright , K. E. Meiklejohn , S. L. States , and S. V. Sollecito . 2005. Little effect of extrapair paternity on the opportunity for sexual selection in Savannah Sparrows (*Passerculus sandwichensis*). Evolution; International Journal of Organic Evolution 59:422–430. 15807426

[evo14473-bib-0024] Germain, R. R. , M. T. Hallworth , S. A. Kaiser , T. S. Sillett , and M. S. Webster . 2021. Variance in within‐pair reproductive success influences the opportunity for selection annually and over the lifetimes of males in a multibrooded songbird. Evolution; International Journal of Organic Evolution 75:915–930. 33433909 10.1111/evo.14166

[evo14473-bib-0025] Griffiths, R. , M. C. Double , K. Orr , and R. J. Dawson . 1998. A DNA test to sex most birds. Molecular Ecology 7:1071–1075. 9711866 10.1046/j.1365-294x.1998.00389.x

[evo14473-bib-0026] Grunst, A. S. , M. L. Grunst , M. L. Korody , L. M. Forrette , R. A. Gonser , and E. M. Tuttle . 2019. Extrapair mating and the strength of sexual selection: insights from a polymorphic species. Behavioral Ecology 30:278–290. 30971857 10.1093/beheco/ary160 PMC6450205

[evo14473-bib-0027] Hadfield, J. D. , D. S. Richardson , and T. Burke . 2006. Towards unbiased parentage assignment: combining genetic, behavioural and spatial data in a Bayesian framework. Molecular Ecology 15:3715–3730. 17032269 10.1111/j.1365-294X.2006.03050.x

[evo14473-bib-0028] Hammers, M. , and L. Brouwer . 2017. Rescue behaviour in a social bird: removal of sticky ‘bird‐catcher tree’ seeds by group members. Behaviour 154:403–411.

[evo14473-bib-0029] Hammers, M. , S. A. Kingma , K. Bebbington , J. van de Crommenacker , L. G. Spurgin , D. S. Richardson , T. Burke , H. L. Dugdale , and J. Komdeur . 2015. Senescence in the wild: insights from a long‐term study on Seychelles warblers. Experimental Gerontology 71:69–79. 26344178 10.1016/j.exger.2015.08.019

[evo14473-bib-0030] Hammers, M. , S. A. Kingma , L. G. Spurgin , K. Bebbington , H. L. Dugdale , T. Burke , J. Komdeur , and D. S. Richardson . 2019. Breeders that receive help age more slowly in a cooperatively breeding bird. Nature Communications 10:1301. 10.1038/s41467-019-09229-3 PMC6428877 30899016

[evo14473-bib-0031] Hammers, M. , D. S. Richardson , T. Burke , and J. Komdeur . 2013. The impact of reproductive investment and early‐life environmental conditions on senescence: support for the disposable soma hypothesis. Journal of Evolutionary Biology 26:1999–2007. 23961923 10.1111/jeb.12204

[evo14473-bib-0032] Harrison, X. A. 2014. Using observation‐level random effects to model overdispersion in count data in ecology and evolution. PeerJ 2:e616 10.7717/peerj.616 25320683 PMC4194460

[evo14473-bib-0033] Herényi, M. , H. Gergely , H. László , Z. Garamszegi , R. Hargitai , G. Michl , B. Rosivall , and J. Török . 2012. Lifetime offspring production in relation to breeding lifespan, attractiveness, and mating status in male collared flycatchers. Oecologia 170:935–942. 22644049 10.1007/s00442-012-2362-4

[evo14473-bib-0034] Hsu, Y. H. , J. Schroeder , I. Winney , T. Burke , and S. Nakagawa . 2015. Are extra‐pair males different from cuckolded males? A case study and a meta‐analytic examination. Molecular Ecology 24:1558–1571. 25706253 10.1111/mec.13124

[evo14473-bib-0035] Hsu, Y. H. , M. J. P. Simons , J. Schroeder , A. Girndt , I. S. Winney , T. Burke , and S. Nakagawa . 2017. Age‐dependent trajectories differ between within‐pair and extra‐pair paternity success. Journal of Evolutionary Biology 30:951–959. 28235138 10.1111/jeb.13058

[evo14473-bib-0036] Isvaran, K. , and S. Sankaran . 2017. Do extra‐group fertilizations increase the potential for sexual selection in male mammals? Biology Letters 13:20170313. 29070588 10.1098/rsbl.2017.0313 PMC5665768

[evo14473-bib-0037] Jennions, M. D. , and M. Petrie . 2000. Why do females mate multiply? A review of the genetic benefits. Biological Reviews of the Cambridge Philosophical Society 75:21–64. 10740892 10.1017/s0006323199005423

[evo14473-bib-0038] Jones, A. G. , and N. L. Ratterman . 2009. Mate choice and sexual selection: what have we learned since Darwin? Proceedings of the National Academy of Sciences 106:10001–10008. 10.1073/pnas.0901129106 PMC2702796 19528643

[evo14473-bib-0039] Jones, A. G. , D. E. Walker , C. Kvarnemo , K. Lindström , and J. C. Avise . 2001. How cuckoldry can decrease the opportunity for sexual selection: data and theory from a genetic parentage analysis of the sand goby, *Pomatoschistus minutus* . Proceedings of the National Academy of Sciences 98:9151–9156. 10.1073/pnas.171310198 PMC55388 11481481

[evo14473-bib-0040] Kingma, S. A. , K. Bebbington , M. Hammers , D. S. Richardson , and J. Komdeur . 2016a. Delayed dispersal and the costs and benefits of different routes to independent breeding in a cooperatively breeding bird. Evolution: International Journal of Organic Evolution 70:2595–2610. 27641712 10.1111/evo.13071 PMC5132126

[evo14473-bib-0041] Kingma, S. A. , J. Komdeur , M. Hammers , and D. S. Richardson . 2016b. The cost of prospecting for dispersal opportunities in a social bird. Biology Letters 12:20160316. 27330175 10.1098/rsbl.2016.0316 PMC4938056

[evo14473-bib-0042] Kirkpatrick, M. , T. Price , and S. J. Arnold . 1990. The Darwin‐Fisher theory of sexual selection in monogamous birds. Evolution; International Journal of Organic Evolution 44:180–193. 28568205 10.1111/j.1558-5646.1990.tb04288.x

[evo14473-bib-0043] Kleven, O. , F. Jacobsen , R. Izadnegahdar , R. J. Robertson , and J. T. Lifjeld . 2006. Male tail streamer length predicts fertilization success in the North American barn swallow (*Hirundo rustica erythrogaster*). Behavioral Ecology and Sociobiology 59:412–418.

[evo14473-bib-0044] Koenig, W. D. , and S. S. Albano . 1987. Lifetime reproductive success, selection and the opportunity for selection in the White‐tailed skimmer *Plathemis lydia* (Odonata: *Libellulidae*). Evolution; Internation Journal of Organic Evolution 41:22–36. 10.1111/j.1558-5646.1987.tb05768.x 28563767

[evo14473-bib-0045] Koenig, W. D. , S. S. Albano , and J. L. Dickinson . 1991. A comparison of methods to partition selection acting via components of fitness: do larger male bullfrogs have greater hatching success? Journal of Evolutionary Biology 4:309–320.

[evo14473-bib-0046] Komdeur, J. 1991. Cooperative breeding in the Seychelles warbler. Cambridge, UK: University of Cambridge.

[evo14473-bib-0047] Komdeur, J. 1992. Importance of habitat saturation and territory quality for evolution of cooperative breeding in the Seychelles warbler. Nature 358:493–495.

[evo14473-bib-0048] Komdeur, J. 1994. Experimental evidence for helping and hindering by previous offspring in the cooperative‐breeding Seychelles warbler *Acrocephalus sechellensis* . Behavioral Ecology and Sociobiology 34:175–186.

[evo14473-bib-0049] Komdeur, J. 2001. Mate guarding in the Seychelles warbler is energetically costly and adjusted to paternity risk. Proceedings of the Royal Society B: Biological Sciences 268:2103–2111. 10.1098/rspb.2001.1750 PMC1088854 11600074

[evo14473-bib-0050] Komdeur, J. , T. Burke , H. L. Dugdale , and D. S. Richardson . 2016. Seychelles warblers: the complexities of the helping paradox. *In* W. D. Koenig and J. L. Dickinson , eds. Cooperative breeding in vertebrates: studies of ecology, evolution, and behavior. Cambridge, UK: Cambridge University Press, pp. 197–217.

[evo14473-bib-0051] Komdeur, J. , A. Huffstadt , W. Prast , G. Castle , R. Mileto , and J. Wattel . 1995. Transfer experiments of Seychelles warblers to new islands: changes in dispersal and helping behaviour. Animal Behaviour 49:695–708.

[evo14473-bib-0052] Komdeur, J. , F. Kraaijeveld‐Smit , K. Kraaijeveld , and P. Edelaar . 1999. Explicit experimental evidence for the role of mate guarding in minimizing loss of paternity in the Seychelles warbler. Proc. R. Soc. B 266:2075–2081.

[evo14473-bib-0053] Komdeur, J. , T. Piersma , K. Kraaijeveld , F. Kraaijeveld‐Smit , and D. S. Richardson . 2004. Why Seychelles Warblers fail to recolonize nearby islands: unwilling or unable to fly there? Ibis 146:298–302.

[evo14473-bib-0054] Lebigre, C. , P. Arcese , and J. M. Reid . 2013. Decomposing variation in male reproductive success: age‐specific variances and covariances through extra‐pair and within‐pair reproduction. Journal of Animal Ecology 82:872–883. 23470041 10.1111/1365-2656.12063

[evo14473-bib-0055] Lebigre, C. , P. Arcese , R. J. Sardell , L. F. Keller , and J. M. Reid . 2012. Extra‐pair paternity and the variance in male fitness in song sparrows (*Melospiza melodia*). Evolution; International Journal of Organic Evolution 66:3111–3129. 23025602 10.1111/j.1558-5646.2012.01677.x

[evo14473-bib-0056] Leclaire, S. , J. F. Nielsen , S. P. Sharp , and T. H. Clutton‐Brock . 2013. Mating strategies in dominant meerkats: evidence for extra‐pair paternity in relation to genetic relatedness between pair mates. Journal of Evolutionary Biology 26:1499–1507. 23675879 10.1111/jeb.12151

[evo14473-bib-0057] Lee‐Jenkins, S. S. Y. , M. L. Smith , B. D. Wisenden , A. Wong , and J.‐G. J. Godin . 2015. Genetic evidence for mixed broods and extra‐pair matings in a socially monogamous biparental cichlid fish. Behaviour 152:1507–1526.

[evo14473-bib-0058] Lemaître, J.‐F. , and J.‐M. Gaillard . 2017. Reproductive senescence: new perspectives in the wild. Biology Reviews 92:2182–2199. 10.1111/brv.12328 28374548

[evo14473-bib-0059] Merilä, J. , and B. C. Sheldon . 2000. Lifetime reproductive success and heritability in nature. Source Am. Nat 155:301–310. 10.1086/303330 10718727

[evo14473-bib-0060] Nakagawa, S. , J. Schroeder , and T. Burke . 2015. Sugar‐free extrapair mating: a comment on Arct et al. Behavioral Ecology 26:971–972.

[evo14473-bib-0061] Nussey, D. H. , H. Froy , J.‐F. Lemaitre , J.‐M. Gaillard , and S. N. Austad . 2013. Senescence in natural populations of animals: widespread evidence and its implications for bio‐gerontology. Ageing Research Reviews 12:214–225. 22884974 10.1016/j.arr.2012.07.004 PMC4246505

[evo14473-bib-0062] Powell, M. J. D. 2009. The BOBYQA algorithm for bound constrained optimization without derivatives. Cambridge, UK: University of Cambridge.

[evo14473-bib-0063] Price, T. D. 1984. Sexual selection on body size, territory and plumage variables in a population of Darwin's finches. Evolution; International Journal of Organic Evolution 38:327–341. 28555895 10.1111/j.1558-5646.1984.tb00291.x

[evo14473-bib-0064] Raj Pant, S. , M. Hammers , J. Komdeur , T. Burke , H. L. Dugdale , and D. S. Richardson . 2020. Age‐dependent changes in infidelity in Seychelles warblers. Molecular Ecology 29:3731–3746. 32706433 10.1111/mec.15563

[evo14473-bib-0065] Raj Pant, S. , J. Komdeur , T. Burke , H. L. Dugdale , and D. S. Richardson . 2019. Socio‐ecological conditions and female infidelity in the Seychelles warbler. Behavioral Ecology 30:1254–1264. 31579133 10.1093/beheco/arz072 PMC6765383

[evo14473-bib-0066] Raj Pant, S. , M. A. Versteegh , M. Hammers , T. Burke , H. L. Dugdale , D. S. Richardson , and J. Komdeur . 2022. Data for: The contribution of extra‐pair paternity to the variation in lifetime and age‐specific male reproductive success in a socially monogamous species. Dryad Digital Repository. 10.5061/dryad.dfn2z350d PMC9322416 35325482

[evo14473-bib-0067] Richardson, D. S. , R. Bristol , and N. J. Shah . 2006. Translocation of Seychelles warbler *Acrocephalus sechellensis* to establish a new population on Denis Island, Seychelles. Conservation Evidence 3:54–57.

[evo14473-bib-0068] Richardson, D. S. , T. Burke , and J. Komdeur . 2002. Direct benefits and the evolutionof female‐biased cooperative breeding in Seychelles warblers. Evolution; International Journal of Organic Evolution 56:2313–2321. 12487360 10.1111/j.0014-3820.2002.tb00154.x

[evo14473-bib-0069] Richardson, D. S. , F. L. Jury , K. Blaakmeer , J. Komdeur , and T. Burke . 2001. Parentage assignment and extra‐group paternity in a cooperative breeder: the Seychelles warbler (*Acrocephalus sechellensis*). Molecular Ecology 10:2263–2273. 11555268 10.1046/j.0962-1083.2001.01355.x

[evo14473-bib-0070] Richardson, D. S. , J. Komdeur , and T. Burke . 2003. Avian behaviour: altruism and infidelity among warblers. Nature 422:580. 10.1038/422580a 12686989

[evo14473-bib-0071] Richardson, D. S. , J. Komdeur , T. Burke , and T. von Schantz . 2005. MHC‐based patterns of social and extra‐pair mate choice in the Seychelles warbler. Proceedings of the Royal Society B: Biological Sciences 272:759–767. 10.1098/rspb.2004.3028 PMC1602051 15870038

[evo14473-bib-0072] Schlicht, E. , and B. Kempenaers . 2013. Effects of social and extra‐pair mating on sexual selection in blue tits (*Cyanistes caeruleus*). Evolution; International Journal of Organic Evolution 67:1420–1434. 23617918 10.1111/evo.12073

[evo14473-bib-0073] Sheldon, B. C. , and H. Ellegren . 1999. Sexual selection resulting from extrapair paternity in collared flycatchers. Animal Behaviour 57:285–298. 10049467 10.1006/anbe.1998.0968

[evo14473-bib-0073a] Sparks, A. M. , L. G. Spurgin , M. van der Velde , E. A. Fairfiled , J. Komdeur , T. Burke , D. S. Richardson and H. L. Dugdale . 2021. Telomere heritability and parental age at conception effects in a wild avian population. Molecular Ecology. 10.1111/mec.15804 33586226

[evo14473-bib-0074] Spurgin, L. G. , D. J. Wright , M. van der Velde , N. J. Collar , J. Komdeur , T. Burke , and D. S. Richardson . 2014. Museum DNA reveals the demographic history of the endangered Seychelles warbler. Applications of Evolution 7:1134–1143. 10.1111/eva.12191 PMC4231601 25553073

[evo14473-bib-0075] Uller, T. , and M. Olsson . 2008. Multiple paternity in reptiles: patterns and processes. Molecular Ecology 17:2566–2580. 18452517 10.1111/j.1365-294X.2008.03772.x

[evo14473-bib-0076] Vindenes, Y. , S. Engen , and B.‐E. Saether . 2008. Individual heterogeneity in vital parameters and demographic stochasticity. Am. Nat 171:455–467. 20374136 10.1086/528965

[evo14473-bib-0077] Weatherhead, P. J. , and P. T. Boag . 1997. Genetic estimates of annual and lifetime reproductive success in male red‐winged blackbird. Ecology 78:884–896.

[evo14473-bib-0078] Webster, M. S. , H. C. Chuang‐Dobbs , and R. T. Holmes . 2001. Microsatellite identification of extrapair sires in a socially monogamous warbler. Behavioral Ecology 12:439–446.

[evo14473-bib-0079] Webster, M. S. , S. Pruett‐Jones , D. F. Westneat , and S. J. Arnold . 1995. Measuring the effects of pairing success, extra‐pair copulations and mate quality on the opportunity for sexual selection. Evolution; International Journal of Organic Evolution 49:1147–1157. 28568519 10.1111/j.1558-5646.1995.tb04441.x

[evo14473-bib-0080] Webster, M. S. , K. A. Tarvin , E. M. Tuttle , and S. Pruett‐Jones . 2007. Promiscuity drives sexual selection in a social monogamous bird. Evolution; International Journal of Organic Evolution 61:2205–2211. 17725624 10.1111/j.1558-5646.2007.00208.x

[evo14473-bib-0081] Westneat, D. F. , and I. R. K. Stewart . 2003. Extra‐pair paternity in birds: causes, correlates, and conflict. Annual Review of Ecology, Evolution, and Systematics 34:365–396.

[evo14473-bib-0082] While, G. M. , T. Uller , and E. Wapstra . 2011. Variation in social organization influences the opportunity for sexual selection in a social lizard. Molecular Ecology 20:844–852. 21199033 10.1111/j.1365-294X.2010.04976.x

[evo14473-bib-0083] Wright, D. J. , L. Brouwer , M.‐E. Mannarelli , T. Burke , J. Komdeur , and D. S. Richardson . 2015. Social pairing of Seychelles warblers under reduced constraints: MHC, neutral heterozygosity, and age. Behavioral Ecology 27:295–303. 26792973 10.1093/beheco/arv150 PMC4718175

[evo14473-bib-0084] Wright, D. J. , N. J. Shah , and D. S. Richardson . 2014. Translocation of the Seychelles warbler *Acrocephalus sechellensis* to establish a new population on Frégate Island, Seychelles. Conservation Evidence 11:20–24.

[evo14473-bib-0085] Yezerinac, S. M. , P. J. Weatherhead , P. T. Boag , J. Weatherhead , P. T. Boag , S. M. Yezerinac , ‐P. J. Weatherhead , and P. T. Boag . 1995. Extra‐pair paternity and the opportunity for sexual selection in a socially monogamous bird (*Dendroica petechia*). Behavioral Ecology and Sociobiology 37:179–188.

