# Supplementary material for: The contribution of extra‐pair paternity to the variation in lifetime and age‐specific male reproductive success in a socially monogamous species
Source: Evolution. 2022 Apr 9;76(5):915–30. doi: 10.1111/evo.14473 (PMC9322416; doi:10.1111/evo.14473)
Supplement: Supplementary file 1 — Figure S1. Distribution of ages at first dominance (years) among Seychelles warbler males who gained a dominant (breeding) position during their life (n = 182). Figure S2. Distribution of lifespan (years) among Seychelles warbler males (n = 237). Figure S3. Additive method: standardized age‐specific (co)variance components of the variance in lifetime reproductive success of male Seychelles warblers (n = 237). Figure S4. Additive method: standardized age‐specific (co)variance components of the variance in the lifetime reproductive success – genetic (A) and social (B) – of male Seychelles warblers (n = 237). Table S1. Additive method: percentage contribution of standardized age‐specific (co)variance components to lifetime reproductive success of male Seychelles warblers (n = 237). Table S2. Decomposition of the variance in lifetime reproductive success (LRS) of male Seychelles warblers that obtained at least one within‐group (WG) and extra‐group (EG) offspring in life (n = 74) into the variance in lifetime within‐ and extra‐group: number of mates (M), female fecundity (number of offspring per mate, N) and paternity allocation (proportion of young sired per mate, P), following Webster et al. (1995). [file EVO-76-915-s001.docx]

**Supplementary Information**


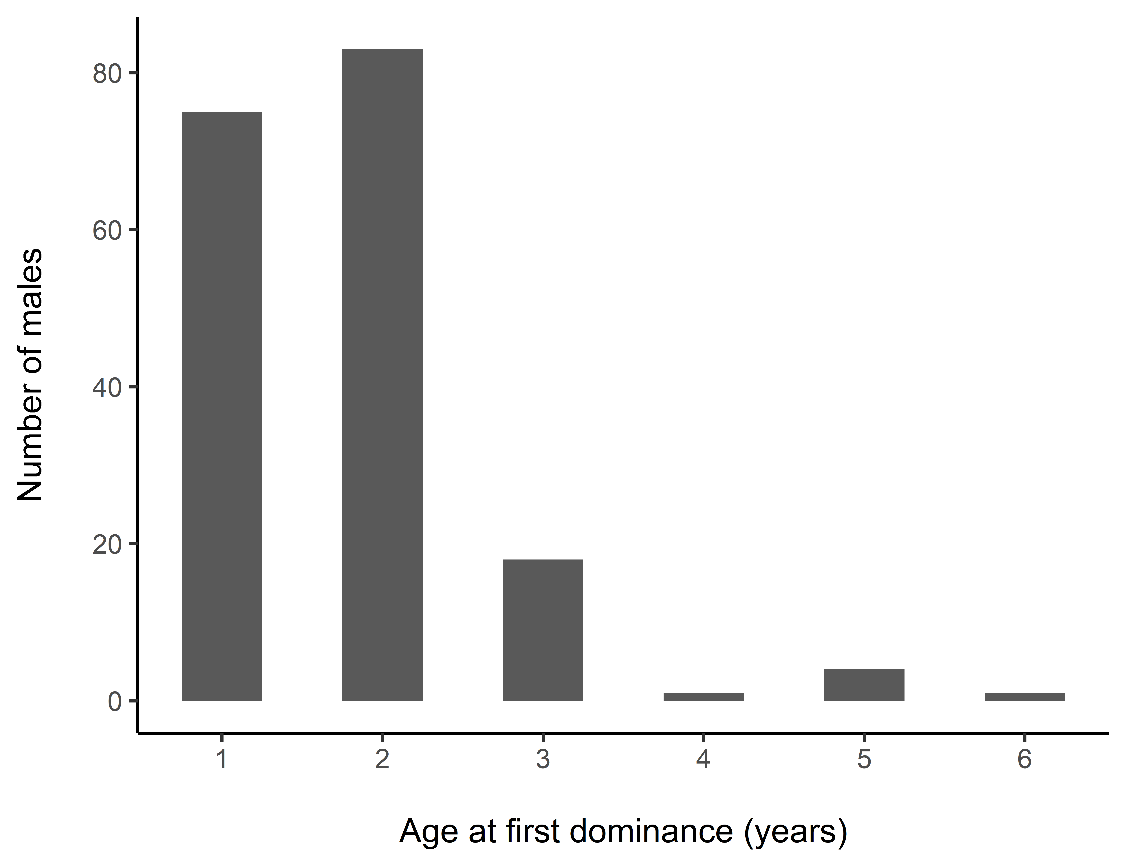


**Figure S1. Distribution of ages at first dominance (years) among Seychelles warbler males who gained a dominant (breeding) position during their life (*n* = 182).**


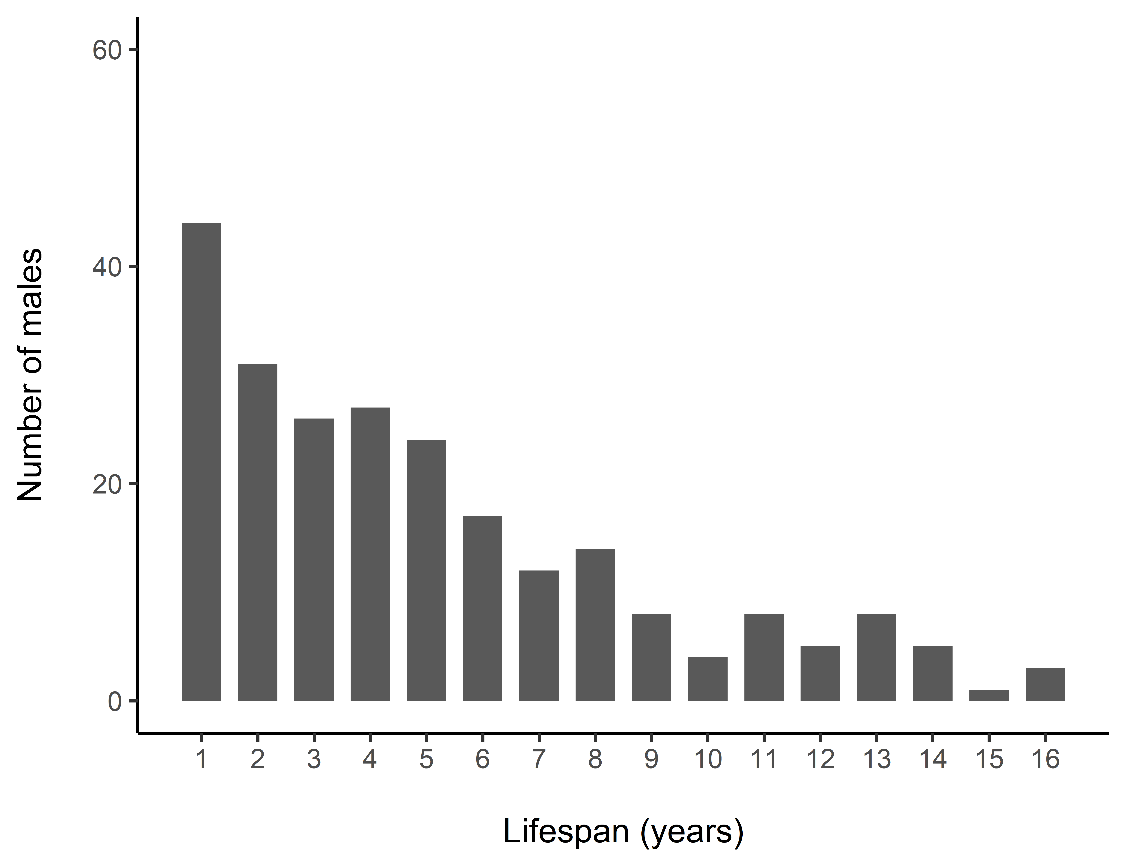


**Figure S2. Distribution of lifespan (years) among Seychelles warbler males (*n* = 237).**


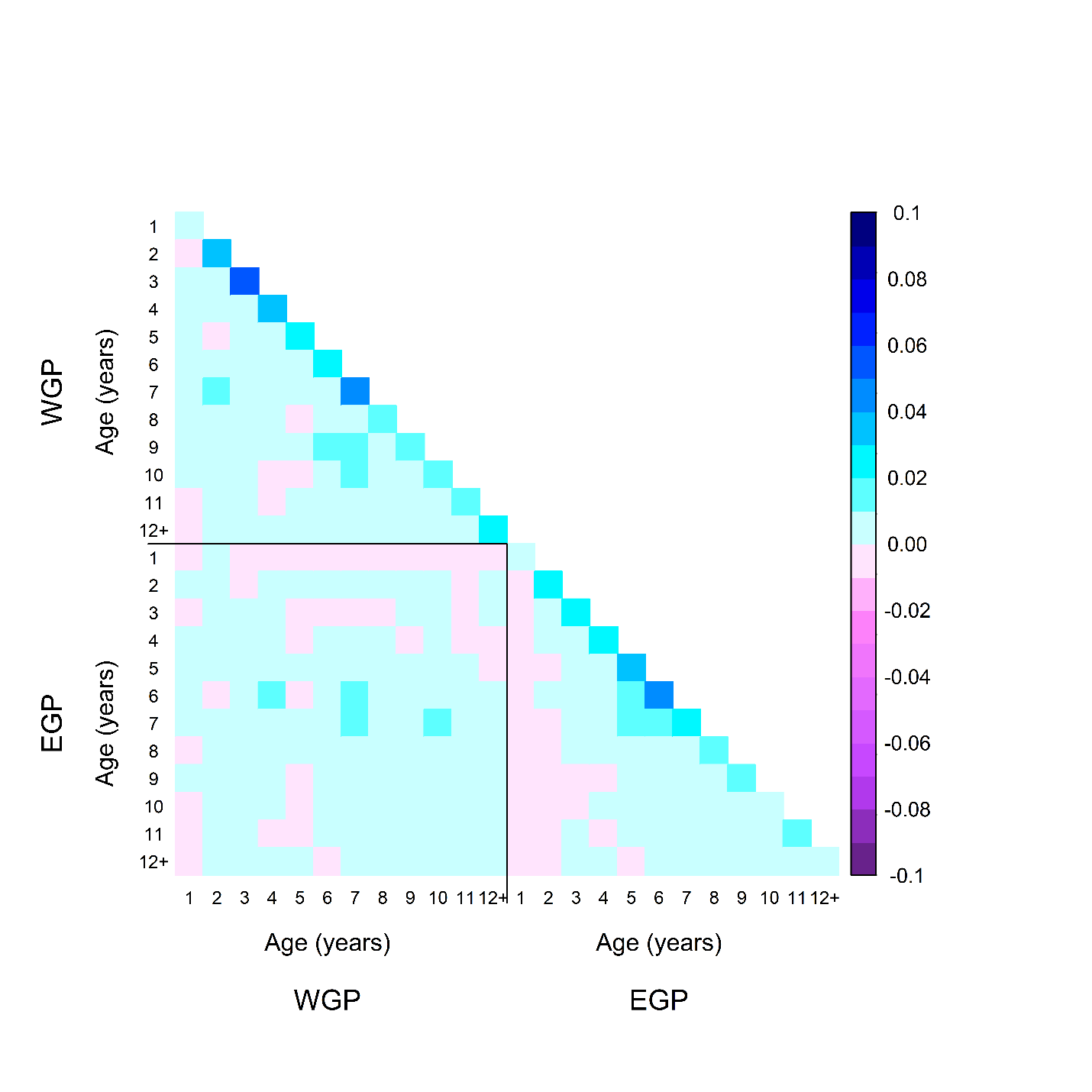


**Figure S3.** **Additive method: standardised age-specific (co)variance components of the variance in lifetime** **reproductive success of male Seychelles warblers (*n* = 237).** (Co)variance values are represented by coloured squares. Shades of blue indicate positive (co)variances, while shades of violet indicate negative (co)variances. Darker squares represent larger (co)variance (absolute) values. Variances in age-specific extra-group paternity (EGP) and within-group paternity (WGP) are found on the diagonal**.** Covariances are found elsewhere on the gird and are: the between-age covariances in WGP (top left triangular panel) and EGP (bottom right triangular panel), and the age-specific (bottom-left of the bottom-left square panel) and between-age (top-right of the bottom-left square panel) covariances between EGP and WGP. All (co)variance components are standardised by dividing by the squared mean of lifetime reproductive success.

**
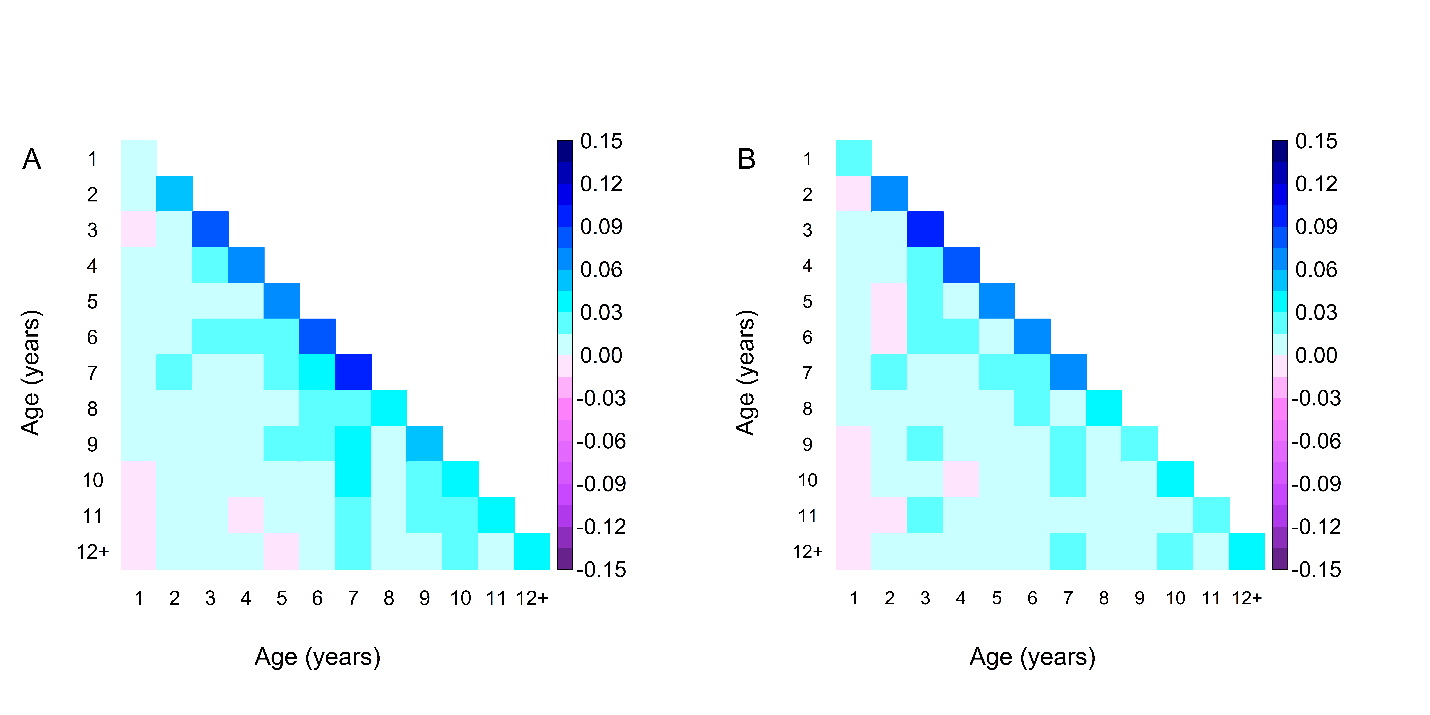
**

**Figure S4.** **Additive method: standardised age-specific (co)variance components of the variance in the lifetime reproductive success – genetic (A) and social (B) – of male Seychelles warblers (*n* = 237).** (Co)variance values are represented by coloured squares. Shades of blue indicate positive (co)variances, while shades of violet indicate negative (co)variances. Darker squares represent larger (co)variance (absolute) values. Variances in age-specific reproductive success are found on the diagonals. Between-age covariances are found elsewhere on the grid. All (co)variance components are standardised by dividing by the squared mean of lifetime genetic or social reproductive success.

**Table S1.** **Additive method: percentage contribution of standardised age-specific (co)variance components to lifetime reproductive success of male Seychelles warblers (*n* = 237).** The variance in lifetime genetic reproductive success (RS) was partitioned into its age-specific components. Variances (Var) in age-specific RS and its components – i.e. age-specific extra-group paternity (EGP), within-group paternity (WGP) and twice the covariances (2Cov) between EGP and WGP – add up to their respective sums of variances or doubled covariances ‘ΣVar_(age-specific)_ or Σ2Cov_(age-specific)_’. Twice the between-age covariances in RS, EGP and WGP, and between EGP and WGP, add up to their respective sums of covariances ‘Σ2Cov_(between-age)_’. The sum of the ΣVar_(age-specific)_ or Σ(2Cov)_(age-specific)_ plus the Σ2Cov_(between-age)_ gives the variances, or doubled covariances, in lifetime paternity success measures, found in the bottom row (‘Lifetime’). The percent contribution to lifetime social (‘apparent’) reproductive success (RS_ap_) of its age-specific (co)variance components are also shown. All (co)variances are standardised by the squared mean of lifetime RS (for genetic paternity measures: EGP, WGP and RS) or lifetime RS_ap_ (for social reproduction).

| Age (years) | % Var(EGP) | % Var(WGP) | % 2Cov(EGP,WGP) | % Var(RS) | % Var(RS_ap_) |
| --- | --- | --- | --- | --- | --- |
| 1 | 0.09 | 0.30 | -0.01 | 0.38 | 1.16 |
| 2 | 0.99 | 1.53 | 0.30 | 2.82 | 3.88 |
| 3 | 1.39 | 2.43 | 0.17 | 3.99 | 5.93 |
| 4 | 1.42 | 1.88 | 0.14 | 3.45 | 4.87 |
| 5 | 1.90 | 1.42 | 0.28 | 3.61 | 3.94 |
| 6 | 1.98 | 1.44 | 0.77 | 4.19 | 3.75 |
| 7 | 1.07 | 2.00 | 1.60 | 4.67 | 4.14 |
| 8 | 0.83 | 0.94 | 0.34 | 2.11 | 2.54 |
| 9 | 0.67 | 0.94 | 0.70 | 2.31 | 1.58 |
| 10 | 0.46 | 0.91 | 0.54 | 1.91 | 2.32 |
| 11 | 0.55 | 0.56 | 0.41 | 1.52 | 1.14 |
| 12-16 | 0.25 | 1.10 | 0.58 | 1.93 | 1.96 |
| Σ Var_(age-specific)_ or Σ2Cov_(age-specific)_ | 11.61 | 15.43 | 5.83 | 32.87 | 37.21 |
| Σ2Cov_(between-age)_ | 14.99 | 23.99 | 28.15 | 67.13 | 62.79 |
| **Lifetime** | **26.60** | **39.42** | **33.98** | **100.00** | **100.00** |

**Table S2.** **Decomposition of the variance in lifetime reproductive success (LRS) of male Seychelles warblers that obtained at least one within-group (WG) and extra-group (EG) offspring in life (*n* = 74) into the variance in lifetime within- and extra-group: number of mates (M), female fecundity (number of offspring per mate, N) and paternity allocation (proportion of young sired per mate, P), following Webster et al. (1995).** Lifetime within-group paternity, ‘LWGP’; lifetime extra-group paternity ‘LEGP’. All (co)variance components are standardised by the squared mean of LRS. Each of the within- and extra-group M, N and P (co)variance terms are calculated using means of additional components (see details on and formulas for the calculation of each term in Webster et al., 1995).

| **Source of variance** | **(Co)variance component** | | **Standardized value** | **% Variance LRS** |
| --- | --- | --- | --- | --- |
| Within-group (WG) | Variance M_WG_ | | 0.11 | 32.74 |
|  | Variance N_WG_ | | 0.13 | 37.51 |
|  | Variance P_WG_ | | 0.06 | 17.97 |
|  | Covariance M_WG_, N_WG_ | | -0.08 | -22.54 |
|  | Covariance M_WG_, P_WG_ | | -0.02 | -5.02 |
|  | Covariance N_WG_, P_WG_ | | -0.05 | -14.25 |
|  | Remainder term | | -0.02 | -7.25 |
|  | **Total LWGP variance** | | **0.13** | **39.14** |
| Extra-group (EG) | Variance M_EG_ | | 0.09 | 26.89 |
|  | Variance N_EG_ | | 0.05 | 13.81 |
|  | Variance P_EG_ | | 0.01 | 2.04 |
|  | Covariance M_EG_, N_EG_ | | -0.002 | -0.47 |
|  | Covariance M_EG_, P_EG_ | | -0.002 | -0.52 |
|  | Covariance N_EG_, P_EG_ | | -0.02 | -4.81 |
|  | Remainder term | | -0.02 | -5.07 |
|  | **Total LEGP variance** | | **0.11** | **31.87** |
| Covariance WG, EG | Covariance M_WG_, M_EG_ | | 0.11 | 33.69 |
|  | Covariance M_WG_, N_EG_ | | -0.02 | -6.39 |
|  | Covariance M_WG_, P_EG_ | | 0.002 | 0.46 |
|  | Covariance N_WG_, M_EG_ | | -0.01 | -3.92 |
|  | Covariance N_WG_, N_EG_ | | 0.03 | 9.23 |
|  | Covariance N_WG_, P_EG_ | | -0.001 | -0.44 |
|  | Covariance P_WG_, M_EG_ | | -0.004 | -1.25 |
|  | Covariance P_WG_, N_EG_ | | 0.01 | 2.68 |
|  | Covariance P_WG_, P_EG_ | | -0.01 | -1.95 |
|  | Remainder term | | -0.01 | -3.13 |
|  | **Total covariance LWGP, LEGP** | | **0.10** | **28.99** |
| **Total variance in LRS** | |  | **0.34** | **100.00** |
