# Supplementary figures and images for: The contribution of extra‐pair paternity to the variation in lifetime and age‐specific male reproductive success in a socially monogamous species
Source: Evolution. 2022 Apr 9;76(5):915–30. doi: 10.1111/evo.14473 (PMC9322416; doi:10.1111/evo.14473)

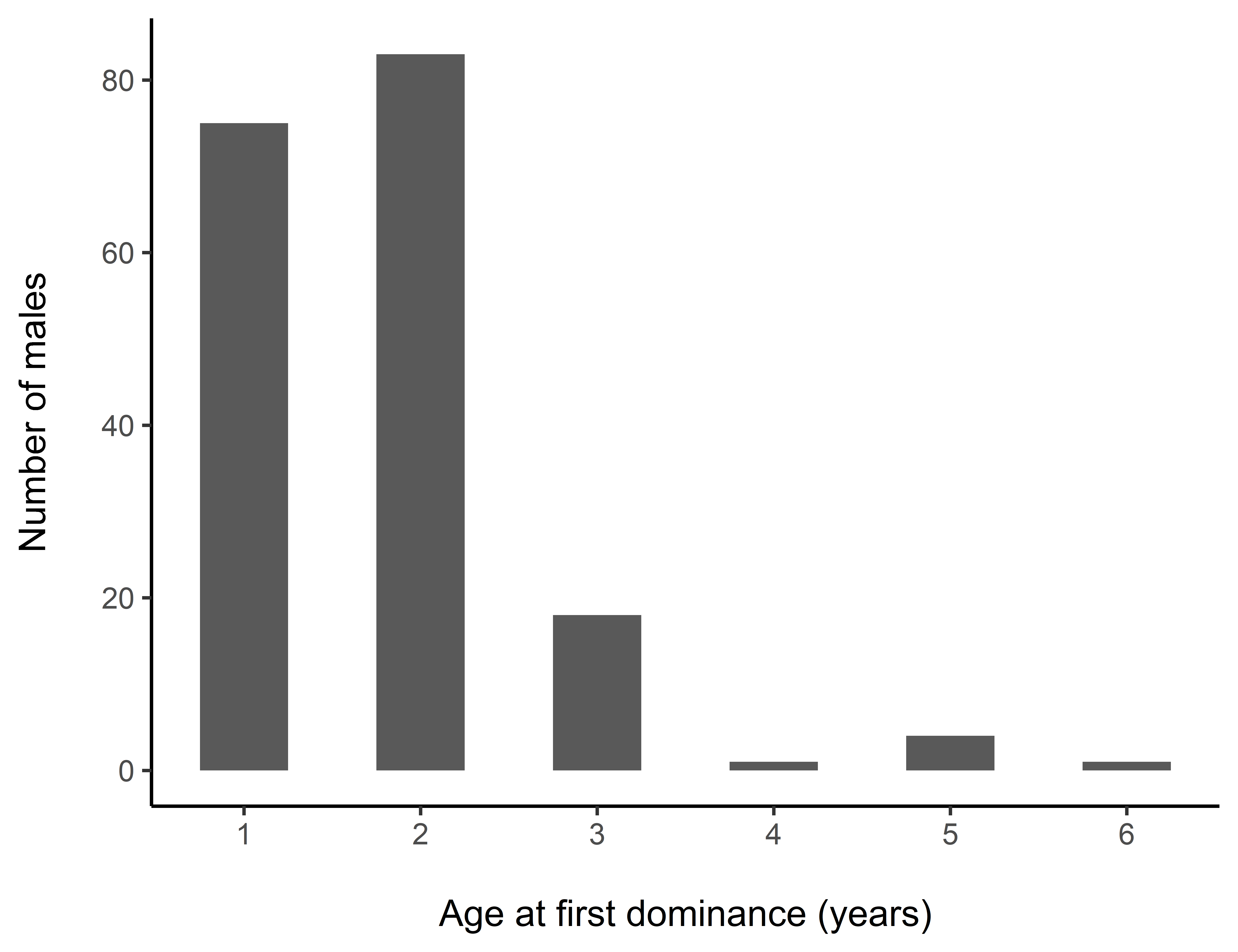

Supplement: Supplementary file 2 — Supplementary information [file EVO-76-915-s005.tiff]

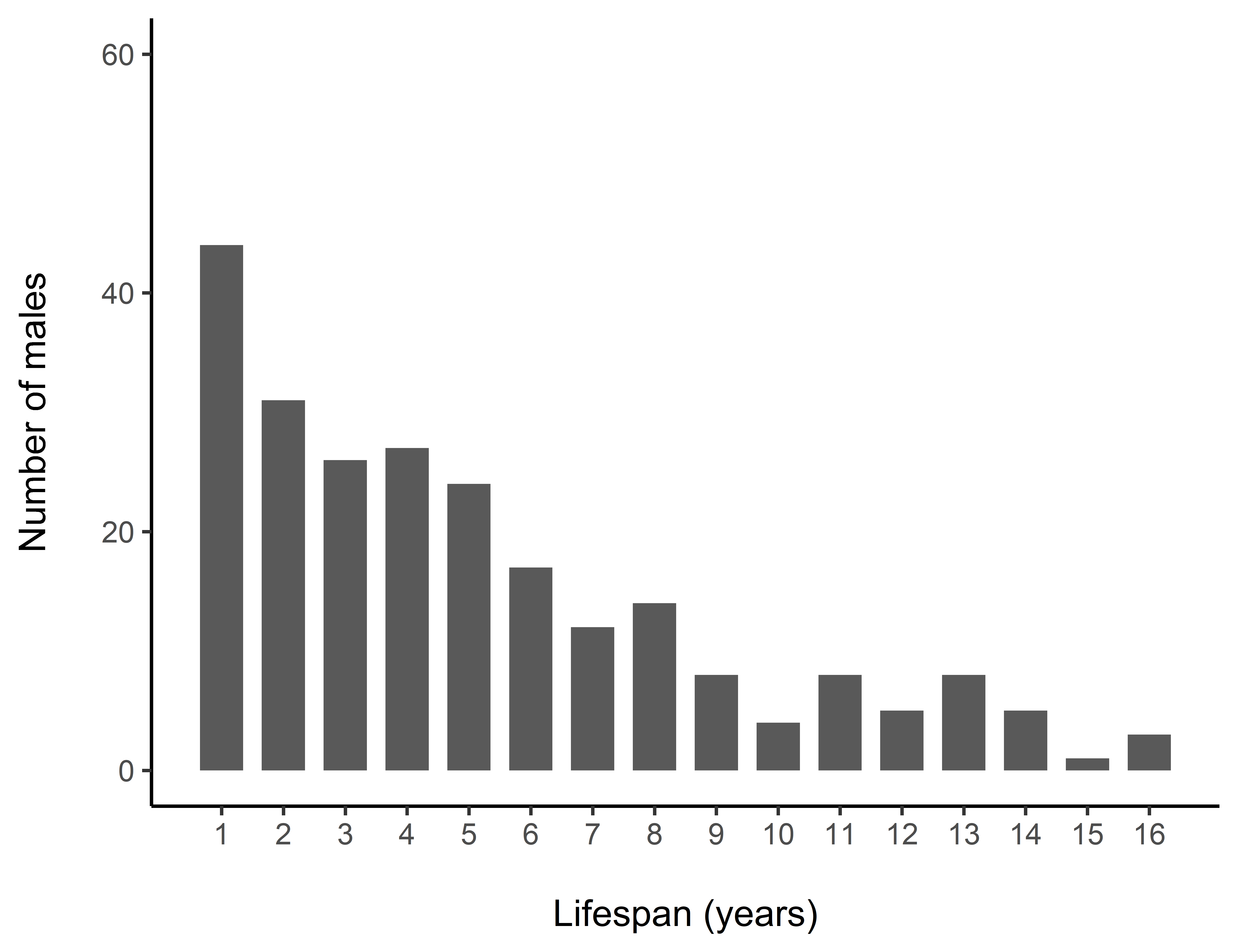

Supplement: Supplementary file 3 — Supplementary information [file EVO-76-915-s004.tiff]
